# The crystal structures and Hirshfeld surface analysis of *N*′,*N*′′′-((1*E*,1′*E*)-{[methyl­enebis(­oxy)]bis­(6-bromo-3,1-phenyl­ene)}bis­(methan­ylyl­idene))bis­(isonicotinohydrazide) dihydrate and *N*′,*N*′′′-((1*E*,1′*E*)-{[butane-1,4-diylbis(­oxy)]bis­(2,1-phenyl­ene)}bis­(methan­ylyl­idene))bis­(isonicotino­hydrazide) [+ solvent]

**DOI:** 10.1107/S2056989019005048

**Published:** 2019-04-18

**Authors:** S. Syed Abuthahir, M. NizamMohideen, V. Viswanathan, Tamilselvan Abiraman, Sengottuvelan Balasubramanian

**Affiliations:** a PG & Research Department of Physics, The New College (Autonomous), Chennai 600 014, Tamil Nadu, India; b Department of Biophysics, All India Institute of Medical Sciences, New Delhi 110 029, India; c Department of Inorganic Chemistry, University of Madras, Chennai 600 025, India

**Keywords:** crystal structure, isonicotino­yl, hydrazide, pyridine, amide, hydrogen bonding, Hirshfeld surface analysis, supra­molecular framework

## Abstract

The title compounds, C_27_H_20_Br_2_N_6_O_4_·2H_2_O, (I), and C_30_H_28_N_6_O_4_·[+ solvent], (II), both crystallize with one half-mol­ecule in the asymmetric unit. The whole mol­ecule of (I) is generated by twofold rotation symmetry, with the twofold rotation axis bis­ecting the C atom of the –O—CH_2_—O– bridge. This results in a folded or U-shaped conformation of the mol­ecule. The whole mol­ecule of (II) is generated by inversion symmetry, with the central CH_2_—CH_2_ bond of the –O—(CH_2_)_4_—O– bridge being located about a center of inversion. This results in a step-like conformation of the mol­ecule. The central C(=O)N—N=C regions of the isonicotinohydrazide moieties in both compounds are planar and the configuration about the imine C=N bonds is *E*. In compound (I), the benzene and pyridine rings are inclined to each other by 37.60 (6)°. The two symmetry-related pyridine rings are inclined to each other by 74.24 (6)°, and the two symmetry-related benzene rings by 7.69 (6)°. In compound (II), the benzene and pyridine rings are inclined to each other by 25.56 (11)°. The symmetry-related pyridine rings are parallel, as are the two symmetry-related benzene rings. In the crystal of (I), a pair of water mol­ecules link the organic mol­ecules *via* O_water_—H⋯O and O_water_—H⋯N hydrogen bonds, forming chains along [001], and enclosing an *R*_4_^2^(8) and two *R*_1_^2^(5) ring motifs. The chains are linked by N—H⋯N_pyridine_ hydrogen bonds, forming a supra­molecular framework. There are also a number of C—H⋯O hydrogen bonds, and C—H⋯π and offset π–π inter­actions [inter­planar distance = 3.294 (1) Å] present reinforcing the framework. In the crystal of (II), mol­ecules are linked by N—H⋯N_pyridine_ hydrogen bonds, forming a supra­molecular framework. Here too there are also a number of C—H⋯O hydrogen bonds present, and a C—H⋯π inter­action, reinforcing the framework. For compound (II), a region of disordered electron density was corrected for using the SQUEEZE [Spek (2015 ▸). Acta Cryst. C71, 9–18] routine in *PLATON*. Their formula mass and unit-cell characteristics were not taken into account during refinement.

## Abstract 2

The title compounds, *N*′,*N*′′′-((1*E*,1′*E*)-{[methyl­enebis(­oxy)]bis­(6-bromo-3,1-phenyl­ene)}bis­(methane­ylyl­idene))bis­(isonicotinohydrazide) dihydrate, (I), and *N*′,*N*′′′-((1*E*,1′*E*)-{[butane-1,4-diylbis(­oxy)]bis­(2,1-phenyl­ene)}bis­(methane­ylyl­idene))bis­(isonicotinohydrazide), (II), both crystallized with half a mol­ecule in the asymmetric unit. The whole mol­ecule of (I) is generated by twofold rotation symmetry, with the twofold rotation axis bis­ecting the C atom of the –O—CH_2_—O– bridge. The whole mol­ecule of (II) is generated by inversion symmetry, with the central CH_2_—CH_2_ bond of the –O—(CH_2_)_4_—O– bridge being located about a center of inversion.

## Chemical context   

Hydrazide-hydrazone compounds are found to be associated with a wide spectrum of biological and medicinal applications. such as anti­microbial, anti­convulsant, analgesic, anti-inflammatory (Kaplancikli *et al.*, 2012 ▸), anti-platelet, anti­bacterial, anti­fungal, anti-tubercular and anti-tumor properties (Babahan *et al.*, 2013 ▸; Bedia *et al.*, 2006 ▸). Schiff bases of the general type *p*-*R*′-C_6_H_4_—CH—N—C_6_H_4_—*R*"-*p* are well-known reagents that find practical application in various areas, *e.g.* photography and medicinal and pharmaceutical chemistry (Sethuram *et al.*, 2013 ▸). Hydrazide Schiff base ligands arise owing to the presence of electron-donating nitro­gen and oxygen atoms, allowing these to act as multidentate ligands, and their transition-metal complexes have been used in the treatment of tuberculosis, in colorimetric or fluorimetric analytic determinations, as well as in applications involving catalytic processes (Torje *et al.*, 2012 ▸) and, in some cases, function as supra­molecular building blocks in their mol­ecular assemblies (Wei *et al.*, 2015 ▸). Hydrazone derivatives containing an azomethine (–CONHN=CH–) group act as cytotoxic agents with the capability to prevent cell series in cancerous cells through different mechanisms (Patil *et al.*, 2011 ▸). Pyridine heterocycles and their derivatives are present in many large mol­ecules having photo-chemical, electrochemical and catalytic applications (Thirunavukkarsu *et al.*, 2017 ▸; Venda *et al.*, 2017 ▸; Jauhar *et al.*, 2016 ▸; Babu *et al.*, 2014*a* ▸,*b* ▸, 2015 ▸; Rajkumar *et al.*, 2014 ▸, 2015 ▸; Huq *et al.*, 2010 ▸). As a part of our research study, we report herein the synthesis and the crystal structures of the title compounds, (I) and (II), which contain several donor functions of a different nature: hydrazide and pyridine.
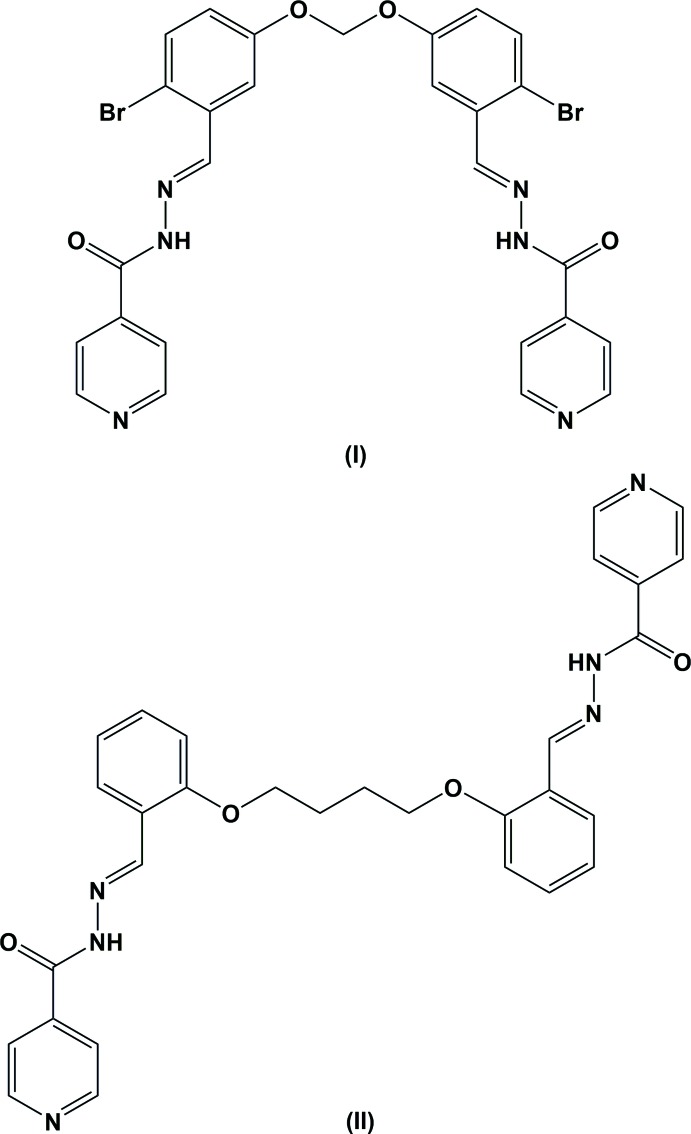


## Structural commentary   

**Figure 1:**
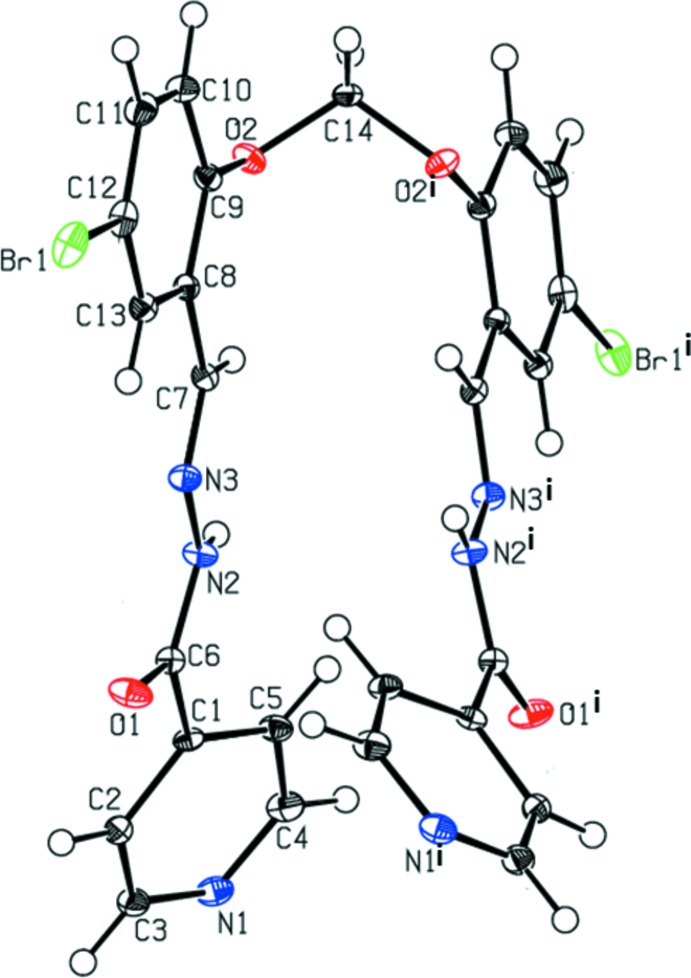
View of the mol­ecular structure of compound (I), with the atom labelling. Displacement ellipsoids are drawn at the 50% probability level. Unlabelled atoms are related to labelled atoms by a twofold rotation axis [symmetry code (i): −*x* + 1, *y*, −*z* + 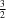]. For clarity, the two water mol­ecules of crystallization have been omitted.

The mol­ecular structures of the title compounds (I) and (II) are illustrated in Figs. 1 ▸ and 2 ▸, respectively. Selected bond lengths and angles are given in Tables 1 ▸ and 2 ▸ for compounds (I) and (II), respectively. The conformations of the two mol­ecules differ considerably. Compound (I) has a folded or U-shaped conformation, while compound (II) has an open step-like conformation. In compound (I), the benzene (C8–C13) and pyridine (N1/C1–C5) rings are inclined to each other by 37.60 (6)°. The two symmetry-related pyridine rings are inclined to each other by 74.24 (6)°, and the two symmetry-related benzene rings by 7.69 (6)°. In compound (II), the benzene and pyridine rings are inclined to each other by 25.56 (11)°. The symmetry-related pyridine rings are parallel, as are the two symmetry-related benzene rings. In both compounds, the hydrazone mol­ecule adopts an *E* configuration with respect to the hydrazone bridge N3=C7, with torsion angle N2—N3—C7—C8 = 176.82 (11) ° in (I) and 179.5 (2)° in (II). On the other hand, torsion angles N3—N2—C6—C1 [−179.8 (1) ° for (I) and 171.5 (2)° for (II)] and C6—N2—N3—C7 [−173.8 (1) ° for (I) and 179.1 (2)° for (II)], are consistent with an *all-trans* relationship in the central chain.

The bond lengths and angles in the carbohydrazide group of the title compounds can be compared with the values reported for related structures (Prabhu *et al.*, 2011 ▸; Bikas *et al.*, 2010 ▸). The N3—N2—C6—O1 torsion angle of 0.2 (2) and −5.6 (3)° for (I) and (II), respectively, indicates the *cis* configuration of the O1 atom with respect to the hydrazine nitro­gen atom N3. The C6—N2 and C7=N3 bond lengths differ by 0.068 (2) Å in (I) and by 0.077 (2) Å in (II), hence these two bonds are properly assigned as single and double bonds, respectively. Bond lengths in the amide unit of aroyl hydrazones are in the ranges 1.218–1.292 Å for C=O bonds and 1.313–1.365 Å for C—N bonds in the keto tautomeric form, and 1.284—1.314 Å for C=O bonds and 1.291–1.331 Å for C—N bonds in the enol tautomeric form (Hosseini-Monfared *et al.*, 2013 ▸). Hence, compounds (I) and (II) are in the keto tautomeric form, which can be verified from the C=O and C—NH bond lengths of the amide unit: O1=C6 [1.230 (2) Å for (I) and 1.223 (2) Å for (II)] and N2—C6 [1.351 (2) Å for (I) and 1.355 (2) Å for (II)]. The bond distances C7=N3 [1.282 (2) Å for (I) and 1.278 (2) Å for (II)] and C6=O1 [1.229 (2) Å for (I) and 1.220 (2) Å for (II)], are very close to the recognized double C=N and C=O bond lengths (Prasanna *et al.*, 2013 ▸; Wang *et al.*, 2010 ▸), confirming that the carbohydrazide exists as an amido tautomer in the solid state. In the two compounds, the three bond angles around atom C6 (see Tables 1 ▸ and 2 ▸) differ from 120°, probably in order to decrease the repulsion between the lone pairs present on atoms N2 and O1.

## Supra­molecular features   

**Figure 3:**
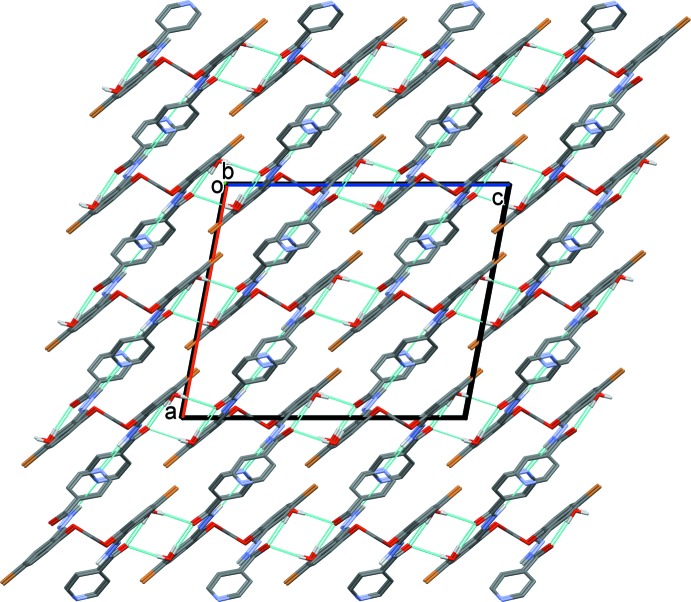
The crystal packing of compound (I), viewed along the *b* axis. The hydrogen bonds are shown as dashed lines (see Table 3 ▸ for details). For clarity, the C-bound H atoms have been omitted.

In the crystal of (I), a pair of water mol­ecules link the organic mol­ecules *via* O_water_—H⋯O and O_water_—H⋯N hydrogen bonds, forming chains along [001] and enclosing an 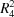
(8) and two 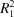
(5) ring motifs (Table 3 ▸ and Fig. 3 ▸). The chains are linked by N—H⋯N_pyridine_ hydrogen bonds, forming a supra­molecular framework. There are also a number of C—H⋯O hydrogen bonds, and C—H⋯π and offset π–π inter­actions [inter­planar distance = 3.294 (1) Å] present, reinforcing the framework (Table 3 ▸). The offset π-π- inter­actions involve inversion-related C8–C13 benzene rings, centroid *Cg*2. The inter­centroid distance *Cg*2⋯*Cg*2(−*x* + 1, −*y* + 1, −*z* + 1) is 3.766 (1) Å, α = 0.00 (6)°, β = 29°, inter­planar distance = 3.294 (1) Å, offset of 1.824 Å.

**Figure 4:**
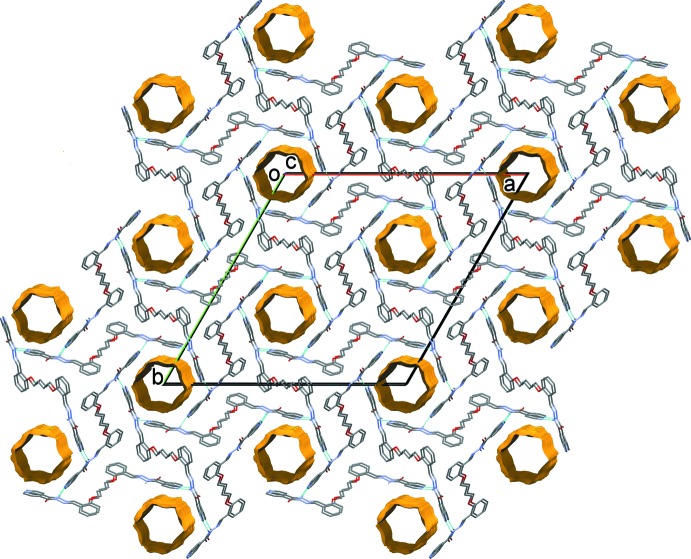
The crystal packing of compound (II), viewed along the *c* axis. The hydrogen bonds are shown as dashed lines (see Table 4 ▸ for details). For clarity, the C-bound H atoms have been omitted. The cylindrical cavities are shown in yellow and brown (*Mercury*; Macrae *et al.*, 2008 ▸).

In the crystal of (II), mol­ecules are linked by N—H⋯N_pyridine_ hydrogen bonds, forming a supra­molecular framework (Table 4 ▸ and Fig. 4 ▸). Here too there are also a number of C—H⋯O hydrogen bonds present, and a C—H⋯π inter­action (Table 4 ▸), reinforcing the framework, but no π–π inter­actions are observed.

For compound (II) a region of disordered electron density with a potential solvent-accessible void of volume 1220 Å^3^ with an electron count of 357 per unit cell was corrected for using the SQUEEZE routine in *PLATON* (Spek, 2015 ▸). The voids in the crystal structure of (II) are illustrated in Fig. 4 ▸.

## Hirshfeld surface analysis   

The Hirshfeld surface analysis (Spackman & Jayatilaka, 2009 ▸), and the associated two-dimensional fingerprint plots (McKinnon *et al.*, 2007 ▸), were calculated to analyse the inter­molecular contacts in the crystals. The various calculations were performed with *CrystalExplorer17* (Turner *et al.*, 2017 ▸). The use of such calculations to analyse inter­molecular contacts in crystals has been reported on recently by Tiekink and collaborators (Tan *et al.*, 2019 ▸).

**Figure 5:**
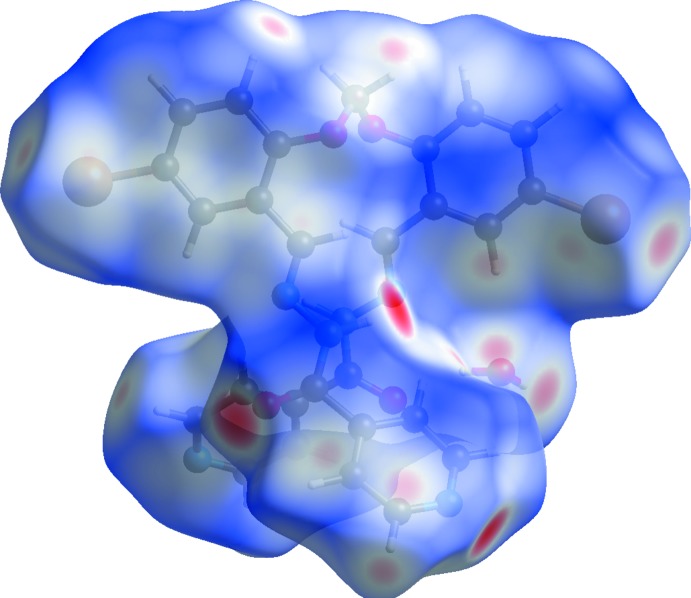
The Hirshfeld surfaces of compound (I), mapped over *d*_norm_; fixed colour scale of −0.512 (red) to 1.285 (blue) arbitrary units.

**Figure 7:**
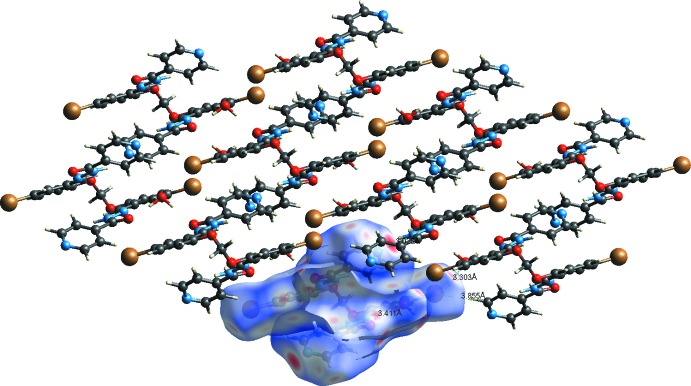
A view of the Hirshfeld surface mapped over *d*_norm_ of compound (I), showing the various inter­molecular contacts in the crystal.

**Figure 8:**
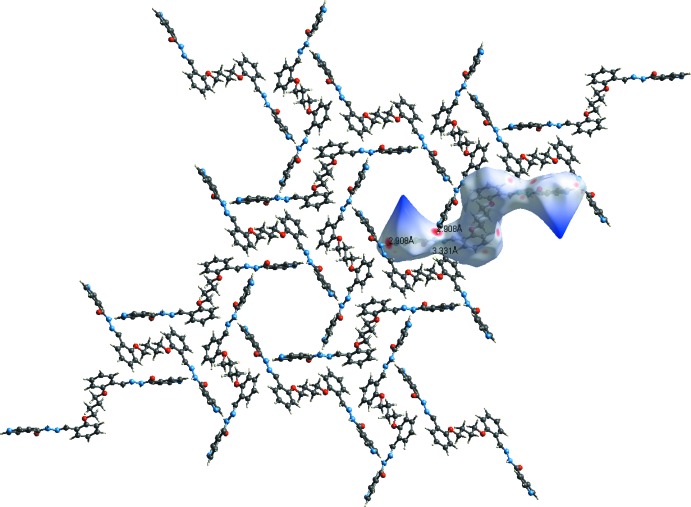
A view of the Hirshfeld surface mapped over *d*_norm_ of compound (II), showing the various inter­molecular contacts in the crystal.

The Hirshfeld surfaces of compounds (I) and (II) mapped over *d*_norm_ are given in Figs. 5 ▸ and 6 ▸, respectively. For (I) the inter­molecular contacts are illustrated in Fig. 7 ▸, and for (II) in Fig. 8 ▸. They are colour-mapped with the normalized contact distance, *d*_norm_, from red (distances shorter than the sum of the van der Waals radii) through white to blue (distances longer than the sum of the van der Waals radii). The *d*_norm_ surface was mapped over a fixed colour scale of −0.512 (red) to 1.285 (blue) for compound (I) and −0.490 (red) to 4.945 (blue) for compound (II), where the red spots indicate the inter­molecular contacts involved in hydrogen bonding (remembering that the disordered solvent in the channels of (II) have been *SQUEEZED* out).

**Figure 9:**
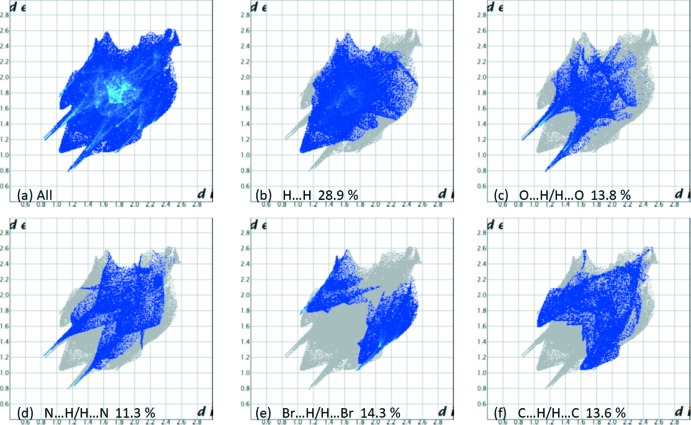
The full two-dimensional fingerprint plot for compound (I), and fingerprint plots delineated into (*b*) H⋯H, (*c*) O⋯H/H⋯O, (*d*) N⋯H/H⋯N, (*e*) Br⋯H/H⋯Br and (*f*) C⋯H/H⋯C contacts.

The fingerprint plots are given in Figs. 9 ▸ and 10 ▸, for compounds (I) and (II), respectively. For compound (I), the principal inter­molecular contacts are H⋯H at 28.9% (Fig. 9 ▸ *b*), O⋯H/H⋯O at 13.8% (Fig. 9 ▸ *c*), N⋯H/H⋯N at 11.3% (Fig. 9 ▸ *d*), Br⋯H/H⋯Br at 14.3% (Fig. 9 ▸ *e*) and C⋯H/H⋯C contacts at 13.6% (Fig. 9 ▸ *f*). C⋯C contacts account for 8.4%, while C⋯Br are 3.0%, C⋯N are 3.0%, and finally C⋯O contacts amount to 1.4%.

**Figure 10:**
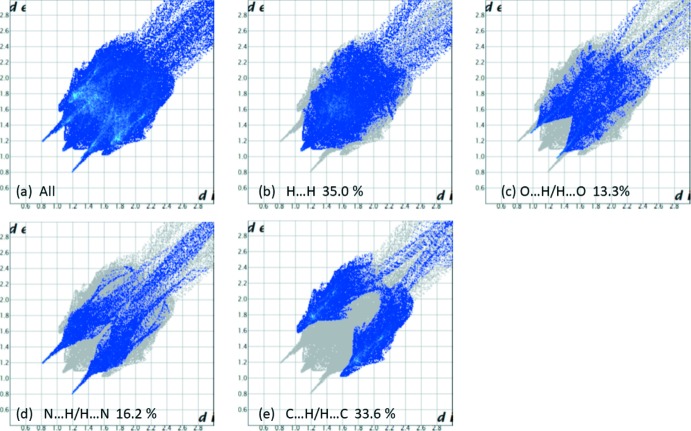
The full two-dimensional fingerprint plot for compound (II), and fingerprint plots delineated into (*b*) H⋯H, (*c*) O⋯H/H⋯O, (*d*) N⋯H/H⋯N, (*e*) C⋯H/H⋯C contacts.

For compound (II), the fingerprint plots reveal that the principal inter­molecular contacts are H⋯H at 35.0% (Fig. 10 ▸ *b*), O⋯H/H⋯O at 13.3% (Fig. 10 ▸ *c*), N⋯H/H⋯N at 16.2% (Fig. 10 ▸ *d*), and C⋯H/H⋯C at 33.6% (Fig. 10 ▸ *e*). The remaining contacts are extremely weak, *ca* 1% each.

## Database survey   

A search of the Cambridge Structural Database (CSD, Version 5.40, February 2019; Groom *et al.*, 2016 ▸) for compounds with an O atom in position 3 of the benzyl­idene ring, *i.e.* (3-*OR*-benzyl­idene)isonicotinohydrazide (*R* = C) skeleton gave 51 hits (supporting information file S1). The majority of these compounds were with an OMe or an OEt substituent.

**Figure 11:**
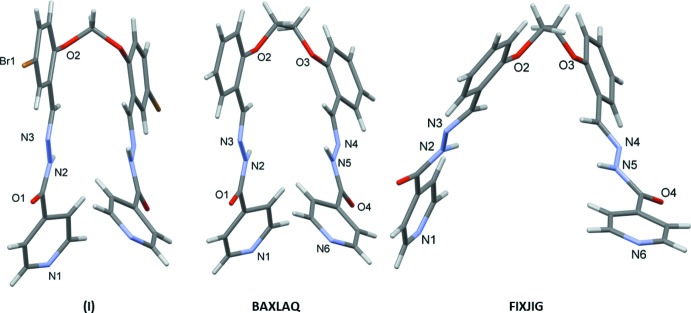
The mol­ecular structures of compounds (I), BAXLAQ (Mahmoudi *et al.*, 2017 ▸) and FIXJIG (Tai *et al.*, 2004 ▸).

A search for compounds with an O atom in position 2 of the benzyl­idene ring, *i.e.* (2-*OR*-benzyl­idene)isonicotinohydrazide (*R* = C) skeleton gave 23 hits (supporting information file S2). Again, the majority of these compounds have an OMe or an OEt substituent. However, here the most inter­esting and relevant compound concerns the ligand *N*′,*N*′′-[ethane-1,2-diylbis(­oxy-2,1-phenyl­ene­methylyl­idene)]bis­(pyridine-4-carbohydrazide), in which a 1,2-di­oxy­ethane bridge links two *N*′-benzyl­ideneisonicotinohydrazide units. The crystal structures of two polymorphs have been described: a monoclinic *P*2_1_ polymorph that crystallizes as a methanol disolvate (BAXLAQ; Mahmoudi *et al.*, 2017 ▸) and a triclinic *P* 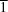 polymorph (FIXJIG; Tai *et al.*, 2004 ▸). The conformation of both compounds is U-shaped, similar to that of compound (I). The mol­ecular structures of compounds (I), BAXLAQ and FIXJIG are compared in Fig. 11 ▸. The principal difference in the conformation of the three mol­ecules is reflected in the dihedral angle involving the benzene rings, which are inclined to each other by 7.69 (6)° in (I), by 25.0 (2)° in BAXLAQ and by 55.27 (7)° in FIXJIG.

An inter­esting HgI_2_ complex of this ligand, bis­(μ-{*N*′,*N*′′-[ethane-1,2-diylbis(­oxy-2,1-phenyl­ene­methylyl­idene)] bis(pyridine-4-carbohydrazide)})tetra­kis­(iodo)­dimercury meth­anol disolvate (BAXKUJ; Mahmoudi *et al.*, 2017 ▸), has a metallamacrocyclic architecture.

## Synthesis and crystallization   

**Compound I**: To 2-hy­droxy­benzaldehyde (5 mmol), in a 250 ml round-bottom (RB) flask was added DMF (30 ml) and potassium carbonate (12.5 mmol). The mixture was stirred at room temperature and then 1,1-di­iodo­butane (2.5 mmol) was added dropwise and the reaction mixture was stirred for 12 h. It was then partitioned between water and ethyl acetate. The ethyl acetate layer was collected and concentrated under reduced pressure. To 1,4-bis­(2-carb­oxy­aldehyde­phen­oxy)butane (2 mmol) and isonicotinic acid hydrazide (4 mmol) in a 250 ml RB flask was added 100 ml of methanol and two drops of glacial acetic acid. The reaction mixture was stirred at room temperature and within 5 min a white-coloured product had formed. The reaction was continued for a further 30 min. The title compound was isolated by filtration and washed with methanol, then chloro­form and followed by acetone. The final product was recrystallized using DMSO and yielded colourless block-like crystals of compound (I).

**Compound I**: To 5-bromo-2-hy­droxy­benzaldehyde (5 mmol), in a 250 ml RB flask, was added 50 ml of DMF and potassium carbonate (12.5 mmol). The mixture was stirred at room temperature and then 1,1-di­iodo­methane (2.5 mmol) was added dropwise. Then, the reaction mixture was stirred for 12 h. The product obtained was extracted in ethyl acetate medium. Methanol (100 ml) and two drops of glacial acetic acid were added to a mixture of 6,6′-[methyl­enebis(­oxy)] bis­(3-bromo­benzaldehyde) (2 mmol) and isoniazid (4 mmol) in a 250 ml RB flask. The reaction mixture was stirred at room temperature and within 5 min a white-coloured product had formed and the reaction was continued for a further 30 min. The solid obtained was washed with methanol, then chloro­form and followed by acetone. The final product was recrystallized using DMSO and yielded colourless block-like crystals of compound (II).

## Refinement   

Crystal data, data collection and structure refinement details are summarized in Table 5 ▸. The NH H atoms for both compounds, and the water mol­ecule H atoms for compound (I), were located in difference-Fourier maps and refined freely. For both compounds the C-bound H atoms were placed in geometrically idealized positions and constrained to ride on their parent atoms: C—H = 0.93-0.97 Å with *U*_iso_(H) = 1.2U_eq_(C).

For compound (II), a region of disordered electron density with a potential solvent accessible void of volume 1220 Å^3^ with an electron count of 357 per unit cell was corrected for using the SQUEEZE routine in *PLATON* (Spek, 2015 ▸). Their formula mass and unit-cell characteristics were not taken into account during refinement.

## Supplementary Material

Crystal structure: contains datablock(s) global, I, II. DOI: 10.1107/S2056989019005048/su5495sup1.cif

Structure factors: contains datablock(s) I. DOI: 10.1107/S2056989019005048/su5495Isup4.hkl

Structure factors: contains datablock(s) II. DOI: 10.1107/S2056989019005048/su5495IIsup5.hkl

Supporting information file. DOI: 10.1107/S2056989019005048/su5495Isup4.cml

Supporting information file. DOI: 10.1107/S2056989019005048/su5495IIsup5.cml

CSD search S1. DOI: 10.1107/S2056989019005048/su5495sup6.pdf

CSD Search S2. DOI: 10.1107/S2056989019005048/su5495sup7.pdf

CCDC references: 1907912, 1907913

Additional supporting information:  crystallographic information; 3D view; checkCIF report

## Figures and Tables

**Figure 2:**
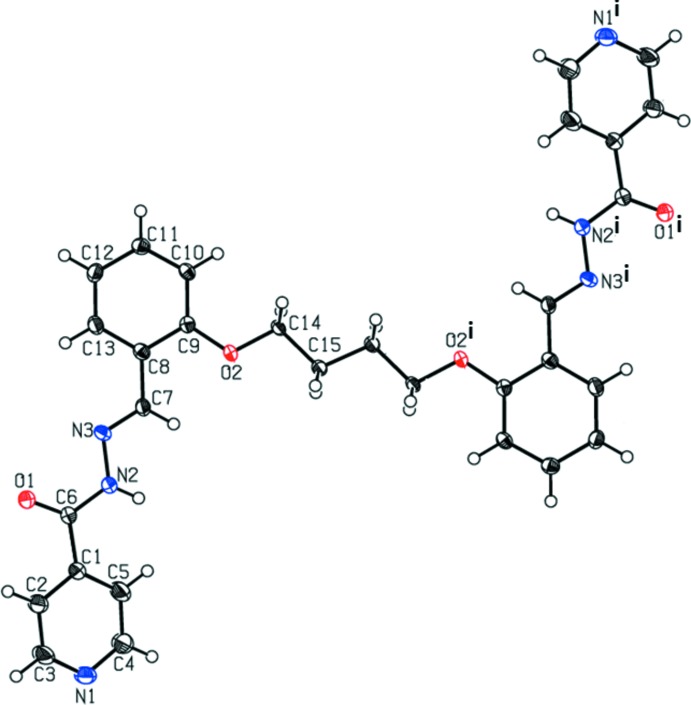
View of the mol­ecular structure of compound (II), with the atom labelling. Displacement ellipsoids are drawn at the 50% probability level. Unlabelled atoms are related to labelled atoms by inversion symmetry [symmetry code (i): −*x*, −*y* + 1, −*z*].

**Figure 6:**
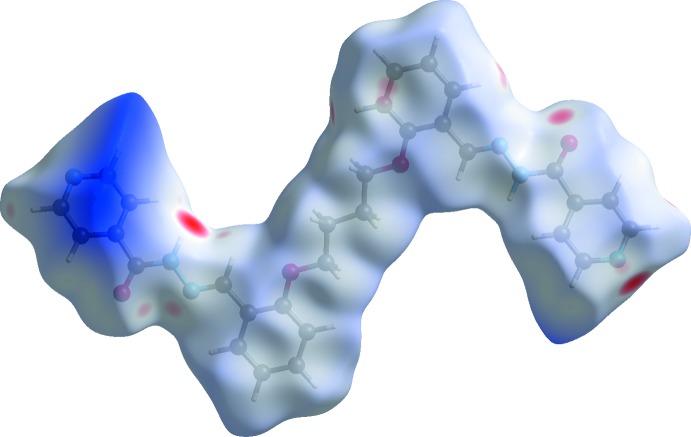
The Hirshfeld surfaces of compound (II), mapped over *d*_norm_; fixed colour scale of −0.490 (red) to 4.945 (blue) arbitrary units.

**Table 1:** Selected geometric parameters (Å, °) for (I)

O1—C6	1.2300 (16)	N3—C7	1.2820 (16)
N2—C6	1.3512 (16)	C1—C6	1.4976 (17)
N2—N3	1.3857 (15)	C7—C8	1.4677 (17)
			
C6—N2—N3	117.46 (11)	O1—C6—C1	120.81 (11)
C7—N3—N2	114.86 (11)	N2—C6—C1	115.38 (11)
O1—C6—N2	123.81 (12)		
			
C6—N2—N3—C7	−173.82 (11)	N3—N2—C6—C1	−179.82 (10)
N3—N2—C6—O1	0.24 (19)	N2—N3—C7—C8	176.82 (11)

**Table 2:** Selected geometric parameters (Å, °) for (II)

O1—C6	1.223 (2)	N3—C7	1.278 (2)
N2—C6	1.355 (2)	C1—C6	1.501 (3)
N2—N3	1.388 (2)	C7—C8	1.463 (3)
			
C6—N2—N3	118.93 (15)	O1—C6—C1	120.65 (16)
C7—N3—N2	115.07 (15)	N2—C6—C1	115.29 (15)
O1—C6—N2	124.00 (17)		
			
C6—N2—N3—C7	179.08 (17)	N3—N2—C6—C1	171.51 (15)
N3—N2—C6—O1	−5.6 (3)	N2—N3—C7—C8	179.54 (15)

**Table 3:** Hydrogen-bond geometry (Å, °) for (I) *Cg*1 is the centroid of N1/C1–C5 pyridine ring.

*D*—H⋯*A*	*D*—H	H⋯*A*	*D*⋯*A*	*D*—H⋯*A*
N2—H*N*2⋯N1^i^	0.85 (2)	2.179 (19)	3.0261 (16)	174 (2)
O1*W*—H1*W*⋯O1	0.86 (2)	2.06 (2)	2.8756 (15)	158 (2)
O1*W*—H1*W*⋯N3	0.86 (2)	2.61 (2)	3.2476 (16)	131.3 (19)
O1*W*—H2*W*⋯O1^ii^	0.83 (3)	2.19 (3)	3.0244 (16)	174 (2)
C3—H3⋯O1*W* ^iii^	0.93	2.56	3.4450 (17)	159
C4—H4⋯Br1^iv^	0.93	2.94	3.8554 (13)	169
C10—H10⋯O1^v^	0.93	2.56	3.4123 (17)	152
C13—H13⋯O1*W*	0.93	2.59	3.5148 (18)	171
C14—H14*A*⋯*Cg*1^v^	0.97	2.74	3.594 (1)	144
C14—H14*B*⋯*Cg*1^vi^	0.97	2.74	3.594 (1)	144

Symmetry codes: (i) 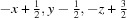; (ii) 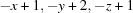; (iii) 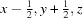; (iv) 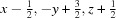; (v) 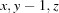; (vi) 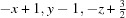.

**Table 4:** Hydrogen-bond geometry (Å, °) for (II) *Cg*2 is the centroid of the C8–C13 benzene ring.

*D*—H⋯*A*	*D*—H	H⋯*A*	*D*⋯*A*	*D*—H⋯*A*
N2—H2*N*⋯N1^i^	0.91 (2)	2.04 (2)	2.907 (2)	159 (2)
C3—H3⋯O1^ii^	0.93	2.60	3.449 (3)	153
C3—H3⋯N3^ii^	0.93	2.55	3.223 (3)	129
C7—H7⋯N1^i^	0.93	2.63	3.372 (3)	137
C12—H12⋯O1^iii^	0.93	2.43	3.331 (2)	163
C15—H15*A*⋯*Cg*2^iv^	0.97	2.91	3.748 (2)	145

Symmetry codes: (i) 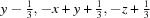; (ii) 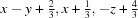; (iii) 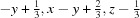; (iv) 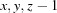.

**Table 5:** Experimental details

	(I)	(II)
Crystal data		
Chemical formula	C_27_H_20_Br_2_N_6_O_4_·2H_2_O	C_30_H_28_N_6_O_4_[+solvent]
*M*_r_	688.34	536.58
Crystal system, space group	Monoclinic, *C*2/*c*	Trigonal, *R* 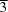 :*H*
Temperature (K)	293	293
*a*, *b*, *c* (Å)	15.1206 (2), 10.1497 (2), 18.0253 (3)	34.3186 (2), 34.3186 (2), 6.7855 (3)
α, β, γ (°)	90, 100.7960 (4), 90	90, 90, 120
*V* (Å^3^)	2717.37 (8)	6921.0 (3)
*Z*	4	9
Radiation type	Mo *K*α	Mo *K*α
μ (mm^−1^)	3.04	0.08
Crystal size (mm)	0.38 × 0.28 × 0.21	0.30 × 0.25 × 0.20

Data collection		
Diffractometer	Bruker Kappa APEXII CCD	Bruker Kappa APEXII CCD
Absorption correction	Multi-scan (*SADABS*; Bruker, 2008 ▸)	Multi-scan (*SADABS*; Bruker, 2008 ▸)
*T*_min_, *T*_max_	0.499, 0.746	0.630, 0.746
No. of measured, independent and observed [*I* > 2σ(*I*)] reflections	29418, 3387, 3202	22262, 3805, 2619
*R*_int_	0.033	0.076
(sin θ/λ)_max_ (Å^−1^)	0.668	0.667

Refinement		
*R*[*F* ^2^ > 2σ(*F* ^2^)], *wR*(*F* ^2^), *S*	0.021, 0.054, 1.05	0.057, 0.142, 1.05
No. of reflections	3387	3805
No. of parameters	199	185
H-atom treatment	H atoms treated by a mixture of independent and constrained refinement	H atoms treated by a mixture of independent and constrained refinement
Δρ_max_, Δρ_min_ (e Å^−3^)	0.49, −0.32	0.47, −0.34

Computer programs: *APEX2* and *SAINT* (Bruker, 2008 ▸), *SHELXS2018* (Sheldrick, 2008 ▸), *SHELXL2018* (Sheldrick, 2015 ▸), *ORTEP-3 for Windows* and *WinGX* (Farrugia, 2012 ▸), *Mercury* (Macrae *et al.*, 2008 ▸), *publCIF* (Westrip, 2010 ▸) and *PLATON* (Spek, 2009 ▸).

## supplementary crystallographic information

### N',N'''-((1E,1'E)-{[Methylenebis(oxy)]bis(6-bromo-3,1-phenylene)}bis(methanylylidene))bis(isonicotinohydrazide) dihydrate (I) Crystal data

**Table:**

C_27_H_20_Br_2_N_6_O_4_·2H_2_O	*F*(000) = 1384
*M**_r_* = 688.34	*D*_x_ = 1.683 Mg m^−^^3^
Monoclinic, *C*2/*c*	Mo *K*α radiation, λ = 0.71073 Å
*a* = 15.1206 (2) Å	Cell parameters from 3387 reflections
*b* = 10.1497 (2) Å	θ = 1.8–26.9°
*c* = 18.0253 (3) Å	µ = 3.04 mm^−^^1^
β = 100.7960 (4)°	*T* = 293 K
*V* = 2717.37 (8) Å^3^	Block, colourless
*Z* = 4	0.38 × 0.28 × 0.21 mm

### N',N'''-((1E,1'E)-{[Methylenebis(oxy)]bis(6-bromo-3,1-phenylene)}bis(methanylylidene))bis(isonicotinohydrazide) dihydrate (I) Data collection

**Table:**

Bruker Kappa APEXII CCD diffractometer	3202 reflections with *I* > 2σ(*I*)
ω and φ scans	*R*_int_ = 0.033
Absorption correction: multi-scan (*SADABS*; Bruker, 2008)	θ_max_ = 28.4°, θ_min_ = 2.8°
*T*_min_ = 0.499, *T*_max_ = 0.746	*h* = −20→20
29418 measured reflections	*k* = −13→13
3387 independent reflections	*l* = −24→24

### N',N'''-((1E,1'E)-{[Methylenebis(oxy)]bis(6-bromo-3,1-phenylene)}bis(methanylylidene))bis(isonicotinohydrazide) dihydrate (I) Refinement

**Table:**

Refinement on *F*^2^	Secondary atom site location: difference Fourier map
Least-squares matrix: full	Hydrogen site location: mixed
*R*[*F*^2^ > 2σ(*F*^2^)] = 0.021	H atoms treated by a mixture of independent and constrained refinement
*wR*(*F*^2^) = 0.054	*w* = 1/[σ^2^(*F*_o_^2^) + (0.0247*P*)^2^ + 3.4271*P*] where *P* = (*F*_o_^2^ + 2*F*_c_^2^)/3
*S* = 1.05	(Δ/σ)_max_ = 0.002
3387 reflections	Δρ_max_ = 0.49 e Å^−^^3^
199 parameters	Δρ_min_ = −0.32 e Å^−^^3^
0 restraints	Extinction correction: (SHELXL2018; Sheldrick, 2015), Fc^*^=kFc[1+0.001xFc^2^λ^3^/sin(2θ)]^-1/4^
Primary atom site location: structure-invariant direct methods	Extinction coefficient: 0.00152 (17)

### N',N'''-((1E,1'E)-{[Methylenebis(oxy)]bis(6-bromo-3,1-phenylene)}bis(methanylylidene))bis(isonicotinohydrazide) dihydrate (I) Special details

**Table:**

Geometry. All esds (except the esd in the dihedral angle between two l.s. planes) are estimated using the full covariance matrix. The cell esds are taken into account individually in the estimation of esds in distances, angles and torsion angles; correlations between esds in cell parameters are only used when they are defined by crystal symmetry. An approximate (isotropic) treatment of cell esds is used for estimating esds involving l.s. planes.

### N',N'''-((1E,1'E)-{[Methylenebis(oxy)]bis(6-bromo-3,1-phenylene)}bis(methanylylidene))bis(isonicotinohydrazide) dihydrate (I) Fractional atomic coordinates and isotropic or equivalent isotropic displacement parameters (Å^2^)

**Table:**

	*x*	*y*	*z*	*U*_iso_*/*U*_eq_	Occ. (<1)
Br1	0.70412 (2)	0.52186 (2)	0.47663 (2)	0.01933 (6)	
O1	0.45281 (7)	1.00457 (9)	0.61053 (6)	0.0176 (2)	
O2	0.45956 (6)	0.35751 (9)	0.68829 (5)	0.01363 (18)	
N1	0.22417 (8)	1.19161 (11)	0.75048 (7)	0.0152 (2)	
N2	0.41385 (7)	0.82530 (10)	0.67331 (6)	0.0115 (2)	
HN2	0.3760 (12)	0.7919 (18)	0.6974 (10)	0.019 (4)*	
N3	0.47208 (7)	0.74664 (10)	0.64190 (6)	0.0120 (2)	
C1	0.34534 (8)	1.03494 (11)	0.68973 (7)	0.0105 (2)	
C2	0.30431 (9)	1.14287 (12)	0.65037 (7)	0.0125 (2)	
H2	0.317244	1.165550	0.603561	0.015*	
C3	0.24360 (9)	1.21635 (13)	0.68217 (7)	0.0144 (2)	
H3	0.214768	1.286668	0.654615	0.017*	
C4	0.26812 (9)	1.09117 (13)	0.78941 (8)	0.0160 (3)	
H4	0.257654	1.074883	0.837801	0.019*	
C5	0.32847 (9)	1.00994 (12)	0.76160 (8)	0.0135 (2)	
H5	0.356824	0.940616	0.790387	0.016*	
C6	0.40899 (9)	0.95414 (12)	0.65410 (7)	0.0111 (2)	
C7	0.46797 (8)	0.62336 (12)	0.65637 (7)	0.0105 (2)	
H7	0.430077	0.593995	0.688011	0.013*	
C8	0.52265 (8)	0.52824 (12)	0.62335 (7)	0.0101 (2)	
C9	0.51576 (8)	0.39410 (12)	0.63961 (7)	0.0114 (2)	
C10	0.55993 (9)	0.29921 (13)	0.60449 (8)	0.0154 (3)	
H10	0.552015	0.210250	0.613749	0.018*	
C11	0.61571 (9)	0.33760 (14)	0.55570 (8)	0.0164 (3)	
H11	0.646693	0.275109	0.532790	0.020*	
C12	0.62469 (9)	0.47104 (13)	0.54149 (7)	0.0136 (2)	
C13	0.57828 (8)	0.56652 (13)	0.57324 (7)	0.0118 (2)	
H13	0.583938	0.654965	0.561477	0.014*	
C14	0.500000	0.27859 (17)	0.750000	0.0157 (4)	
H14A	0.454724	0.222442	0.765344	0.019*	0.5
H14B	0.545278	0.222447	0.734654	0.019*	0.5
O1W	0.60456 (7)	0.90764 (12)	0.55169 (7)	0.0256 (2)	
H1W	0.5551 (16)	0.914 (2)	0.5685 (12)	0.037 (6)*	
H2W	0.5926 (16)	0.934 (2)	0.5072 (14)	0.041 (6)*	

### N',N'''-((1E,1'E)-{[Methylenebis(oxy)]bis(6-bromo-3,1-phenylene)}bis(methanylylidene))bis(isonicotinohydrazide) dihydrate (I) Atomic displacement parameters (Å^2^)

**Table:**

	*U*^11^	*U*^22^	*U*^33^	*U*^12^	*U*^13^	*U*^23^
Br1	0.01383 (9)	0.03079 (10)	0.01546 (8)	−0.00484 (5)	0.00808 (5)	−0.00590 (5)
O1	0.0203 (5)	0.0125 (4)	0.0235 (5)	0.0021 (4)	0.0133 (4)	0.0048 (4)
O2	0.0142 (4)	0.0126 (4)	0.0148 (4)	0.0007 (3)	0.0046 (3)	0.0048 (3)
N1	0.0150 (5)	0.0127 (5)	0.0192 (6)	−0.0003 (4)	0.0067 (4)	−0.0031 (4)
N2	0.0114 (5)	0.0095 (5)	0.0158 (5)	0.0010 (4)	0.0080 (4)	0.0013 (4)
N3	0.0116 (5)	0.0108 (5)	0.0147 (5)	0.0021 (4)	0.0052 (4)	−0.0003 (4)
C1	0.0095 (6)	0.0082 (5)	0.0143 (6)	−0.0016 (4)	0.0035 (4)	−0.0019 (4)
C2	0.0138 (6)	0.0123 (5)	0.0115 (5)	0.0001 (4)	0.0024 (4)	−0.0008 (4)
C3	0.0144 (6)	0.0122 (5)	0.0163 (6)	0.0023 (5)	0.0020 (5)	−0.0008 (5)
C4	0.0204 (7)	0.0135 (6)	0.0167 (6)	−0.0017 (5)	0.0099 (5)	−0.0003 (5)
C5	0.0164 (6)	0.0097 (5)	0.0154 (6)	−0.0013 (4)	0.0055 (5)	0.0015 (4)
C6	0.0104 (6)	0.0106 (5)	0.0125 (6)	0.0000 (4)	0.0027 (4)	0.0000 (4)
C7	0.0091 (5)	0.0116 (5)	0.0108 (5)	0.0003 (4)	0.0023 (4)	0.0008 (4)
C8	0.0080 (6)	0.0104 (5)	0.0112 (6)	0.0006 (4)	0.0003 (4)	0.0000 (4)
C9	0.0098 (6)	0.0117 (5)	0.0124 (5)	0.0006 (4)	0.0012 (4)	0.0012 (4)
C10	0.0176 (6)	0.0112 (6)	0.0169 (6)	0.0028 (5)	0.0020 (5)	−0.0006 (5)
C11	0.0144 (6)	0.0178 (6)	0.0170 (6)	0.0044 (5)	0.0030 (5)	−0.0044 (5)
C12	0.0087 (6)	0.0211 (6)	0.0118 (6)	−0.0007 (5)	0.0036 (5)	−0.0018 (5)
C13	0.0095 (6)	0.0128 (5)	0.0128 (6)	−0.0004 (4)	0.0014 (4)	0.0000 (4)
C14	0.0271 (10)	0.0076 (7)	0.0118 (8)	0.000	0.0019 (7)	0.000
O1W	0.0151 (5)	0.0396 (7)	0.0241 (6)	0.0057 (5)	0.0085 (4)	0.0119 (5)

### N',N'''-((1E,1'E)-{[Methylenebis(oxy)]bis(6-bromo-3,1-phenylene)}bis(methanylylidene))bis(isonicotinohydrazide) dihydrate (I) Geometric parameters (Å, º)

**Table:**

Br1—C12	1.8978 (13)	C4—H4	0.9300
O1—C6	1.2300 (16)	C5—H5	0.9300
O2—C9	1.3818 (16)	C7—C8	1.4677 (17)
O2—C14	1.4134 (13)	C7—H7	0.9300
N1—C4	1.3412 (18)	C8—C13	1.3997 (18)
N1—C3	1.3421 (18)	C8—C9	1.4007 (17)
N2—C6	1.3512 (16)	C9—C10	1.3904 (18)
N2—N3	1.3857 (15)	C10—C11	1.384 (2)
N2—HN2	0.851 (18)	C10—H10	0.9300
N3—C7	1.2820 (16)	C11—C12	1.3897 (19)
C1—C2	1.3869 (17)	C11—H11	0.9300
C1—C5	1.3898 (18)	C12—C13	1.3818 (18)
C1—C6	1.4976 (17)	C13—H13	0.9300
C2—C3	1.3873 (18)	C14—H14A	0.9700
C2—H2	0.9300	C14—H14B	0.9700
C3—H3	0.9300	O1W—H1W	0.86 (2)
C4—C5	1.3910 (19)	O1W—H2W	0.83 (3)
			
C9—O2—C14	115.16 (8)	C8—C7—H7	119.8
C4—N1—C3	116.75 (11)	C13—C8—C9	118.83 (12)
C6—N2—N3	117.46 (11)	C13—C8—C7	122.10 (11)
C6—N2—HN2	120.5 (12)	C9—C8—C7	119.00 (11)
N3—N2—HN2	121.2 (12)	O2—C9—C10	120.53 (11)
C7—N3—N2	114.86 (11)	O2—C9—C8	118.22 (11)
C2—C1—C5	118.58 (12)	C10—C9—C8	121.14 (12)
C2—C1—C6	118.36 (11)	C11—C10—C9	119.76 (12)
C5—C1—C6	123.00 (11)	C11—C10—H10	120.1
C1—C2—C3	118.72 (12)	C9—C10—H10	120.1
C1—C2—H2	120.6	C10—C11—C12	118.94 (12)
C3—C2—H2	120.6	C10—C11—H11	120.5
N1—C3—C2	123.64 (12)	C12—C11—H11	120.5
N1—C3—H3	118.2	C13—C12—C11	122.17 (12)
C2—C3—H3	118.2	C13—C12—Br1	119.57 (10)
N1—C4—C5	123.79 (13)	C11—C12—Br1	118.26 (10)
N1—C4—H4	118.1	C12—C13—C8	119.06 (12)
C5—C4—H4	118.1	C12—C13—H13	120.5
C1—C5—C4	118.37 (12)	C8—C13—H13	120.5
C1—C5—H5	120.8	O2^i^—C14—O2	110.96 (14)
C4—C5—H5	120.8	O2^i^—C14—H14A	109.4
O1—C6—N2	123.81 (12)	O2—C14—H14A	109.4
O1—C6—C1	120.81 (11)	O2^i^—C14—H14B	109.4
N2—C6—C1	115.38 (11)	O2—C14—H14B	109.4
N3—C7—C8	120.47 (11)	H14A—C14—H14B	108.0
N3—C7—H7	119.8	H1W—O1W—H2W	106 (2)
			
C6—N2—N3—C7	−173.82 (11)	N3—C7—C8—C9	−179.26 (12)
C5—C1—C2—C3	−4.17 (18)	C14—O2—C9—C10	57.04 (16)
C6—C1—C2—C3	178.46 (11)	C14—O2—C9—C8	−126.67 (12)
C4—N1—C3—C2	1.2 (2)	C13—C8—C9—O2	−179.05 (11)
C1—C2—C3—N1	2.3 (2)	C7—C8—C9—O2	−2.05 (17)
C3—N1—C4—C5	−2.8 (2)	C13—C8—C9—C10	−2.79 (18)
C2—C1—C5—C4	2.71 (19)	C7—C8—C9—C10	174.21 (12)
C6—C1—C5—C4	179.95 (12)	O2—C9—C10—C11	179.78 (11)
N1—C4—C5—C1	0.9 (2)	C8—C9—C10—C11	3.60 (19)
N3—N2—C6—O1	0.24 (19)	C9—C10—C11—C12	−1.5 (2)
N3—N2—C6—C1	−179.82 (10)	C10—C11—C12—C13	−1.4 (2)
C2—C1—C6—O1	29.80 (18)	C10—C11—C12—Br1	178.26 (10)
C5—C1—C6—O1	−147.44 (13)	C11—C12—C13—C8	2.17 (19)
C2—C1—C6—N2	−150.15 (12)	Br1—C12—C13—C8	−177.48 (9)
C5—C1—C6—N2	32.61 (18)	C9—C8—C13—C12	−0.08 (18)
N2—N3—C7—C8	176.82 (11)	C7—C8—C13—C12	−176.98 (11)
N3—C7—C8—C13	−2.37 (19)	C9—O2—C14—O2^i^	87.86 (9)

Symmetry code: (i) −*x*+1, *y*, −*z*+3/2.

### N',N'''-((1E,1'E)-{[Methylenebis(oxy)]bis(6-bromo-3,1-phenylene)}bis(methanylylidene))bis(isonicotinohydrazide) dihydrate (I) Hydrogen-bond geometry (Å, º)

Cg1 is the centroid of N1/C1–C5 pyridine ring.

**Table:**

*D*—H···*A*	*D*—H	H···*A*	*D*···*A*	*D*—H···*A*
N2—H*N*2···N1^ii^	0.85 (2)	2.179 (19)	3.0261 (16)	174 (2)
O1*W*—H1*W*···O1	0.86 (2)	2.06 (2)	2.8756 (15)	158 (2)
O1*W*—H1*W*···N3	0.86 (2)	2.61 (2)	3.2476 (16)	131.3 (19)
O1*W*—H2*W*···O1^iii^	0.83 (3)	2.19 (3)	3.0244 (16)	174 (2)
C3—H3···O1*W*^iv^	0.93	2.56	3.4450 (17)	159
C4—H4···Br1^v^	0.93	2.94	3.8554 (13)	169
C10—H10···O1^vi^	0.93	2.56	3.4123 (17)	152
C13—H13···O1*W*	0.93	2.59	3.5148 (18)	171
C14—H14*A*···*Cg*1^vi^	0.97	2.74	3.594 (1)	144
C14—H14*B*···*Cg*1^vii^	0.97	2.74	3.594 (1)	144

Symmetry codes: (ii) −*x*+1/2, *y*−1/2, −*z*+3/2; (iii) −*x*+1, −*y*+2, −*z*+1; (iv) *x*−1/2, *y*+1/2, *z*; (v) *x*−1/2, −*y*+3/2, *z*+1/2; (vi) *x*, *y*−1, *z*; (vii) −*x*+1, *y*−1, −*z*+3/2.

### N',N'''-((1E,1'E)-{[Butane-1,4-diylbis(oxy)]\ bis(2,1-phenylene)}bis(methanylylidene))bis(isonicotinohydrazide) (II) Crystal data

**Table:**

C_30_H_28_N_6_O_4_[+solvent]	*D*_x_ = 1.159 Mg m^−^^3^
*M**_r_* = 536.58	Mo *K*α radiation, λ = 0.71073 Å
Trigonal, *R*3:*H*	Cell parameters from 3792 reflections
*a* = 34.3186 (2) Å	θ = 1.8–26.9°
*c* = 6.7855 (3) Å	µ = 0.08 mm^−^^1^
*V* = 6921.0 (3) Å^3^	*T* = 293 K
*Z* = 9	Block, colourless
*F*(000) = 2538	0.30 × 0.25 × 0.20 mm

### N',N'''-((1E,1'E)-{[Butane-1,4-diylbis(oxy)]\ bis(2,1-phenylene)}bis(methanylylidene))bis(isonicotinohydrazide) (II) Data collection

**Table:**

Bruker Kappa APEXII CCD diffractometer	2619 reflections with *I* > 2σ(*I*)
ω and φ scans	*R*_int_ = 0.076
Absorption correction: multi-scan (*SADABS*; Bruker, 2008)	θ_max_ = 28.3°, θ_min_ = 2.1°
*T*_min_ = 0.630, *T*_max_ = 0.746	*h* = −45→36
22262 measured reflections	*k* = −29→45
3805 independent reflections	*l* = −9→9

### N',N'''-((1E,1'E)-{[Butane-1,4-diylbis(oxy)]\ bis(2,1-phenylene)}bis(methanylylidene))bis(isonicotinohydrazide) (II) Refinement

**Table:**

Refinement on *F*^2^	Primary atom site location: structure-invariant direct methods
Least-squares matrix: full	Secondary atom site location: difference Fourier map
*R*[*F*^2^ > 2σ(*F*^2^)] = 0.057	Hydrogen site location: mixed
*wR*(*F*^2^) = 0.142	H atoms treated by a mixture of independent and constrained refinement
*S* = 1.05	*w* = 1/[σ^2^(*F*_o_^2^) + (0.0533*P*)^2^ + 11.1328*P*] where *P* = (*F*_o_^2^ + 2*F*_c_^2^)/3
3805 reflections	(Δ/σ)_max_ < 0.001
185 parameters	Δρ_max_ = 0.47 e Å^−^^3^
0 restraints	Δρ_min_ = −0.34 e Å^−^^3^

### N',N'''-((1E,1'E)-{[Butane-1,4-diylbis(oxy)]\ bis(2,1-phenylene)}bis(methanylylidene))bis(isonicotinohydrazide) (II) Special details

**Table:**

Geometry. All esds (except the esd in the dihedral angle between two l.s. planes) are estimated using the full covariance matrix. The cell esds are taken into account individually in the estimation of esds in distances, angles and torsion angles; correlations between esds in cell parameters are only used when they are defined by crystal symmetry. An approximate (isotropic) treatment of cell esds is used for estimating esds involving l.s. planes.

### N',N'''-((1E,1'E)-{[Butane-1,4-diylbis(oxy)]\ bis(2,1-phenylene)}bis(methanylylidene))bis(isonicotinohydrazide) (II) Fractional atomic coordinates and isotropic or equivalent isotropic displacement parameters (Å^2^)

**Table:**

	*x*	*y*	*z*	*U*_iso_*/*U*_eq_	
O1	0.20009 (4)	0.45231 (5)	0.72823 (18)	0.0262 (3)	
O2	0.02621 (4)	0.46026 (4)	0.34035 (18)	0.0214 (3)	
N1	0.31092 (6)	0.49292 (7)	0.1812 (2)	0.0346 (4)	
N2	0.16142 (5)	0.46016 (5)	0.4732 (2)	0.0218 (3)	
H2N	0.1653 (8)	0.4749 (8)	0.358 (3)	0.033 (6)*	
N3	0.12211 (5)	0.44325 (5)	0.5828 (2)	0.0210 (3)	
C1	0.23686 (6)	0.47415 (6)	0.4186 (3)	0.0223 (4)	
C2	0.27559 (8)	0.47786 (10)	0.4947 (3)	0.0471 (7)	
H2	0.277753	0.474161	0.629269	0.056*	
C3	0.31139 (8)	0.48705 (10)	0.3729 (3)	0.0480 (7)	
H3	0.337126	0.489203	0.428924	0.058*	
C4	0.27280 (9)	0.48779 (11)	0.1061 (3)	0.0538 (8)	
H4	0.271346	0.490922	−0.029363	0.065*	
C5	0.23528 (8)	0.47810 (10)	0.2170 (3)	0.0457 (7)	
H5	0.209289	0.474298	0.156458	0.055*	
C6	0.19813 (6)	0.46179 (6)	0.5561 (3)	0.0201 (4)	
C7	0.08925 (6)	0.44343 (6)	0.4950 (3)	0.0207 (4)	
H7	0.093119	0.454627	0.367374	0.025*	
C8	0.04564 (6)	0.42628 (6)	0.5923 (3)	0.0197 (4)	
C9	0.03514 (7)	0.40109 (6)	0.7662 (3)	0.0240 (4)	
H9	0.056366	0.395193	0.822447	0.029*	
C10	−0.00624 (7)	0.38487 (7)	0.8553 (3)	0.0243 (4)	
H10	−0.012915	0.368208	0.970983	0.029*	
C11	−0.03787 (6)	0.39371 (6)	0.7700 (3)	0.0216 (4)	
H11	−0.065758	0.382899	0.830143	0.026*	
C12	−0.02860 (6)	0.41838 (6)	0.5968 (3)	0.0188 (4)	
H12	−0.050141	0.423846	0.540846	0.023*	
C13	0.01323 (6)	0.43478 (6)	0.5082 (2)	0.0177 (4)	
C14	−0.00685 (6)	0.46611 (6)	0.2364 (2)	0.0178 (4)	
H14A	−0.018719	0.480568	0.320625	0.021*	
H14B	−0.031546	0.437179	0.195219	0.021*	
C15	0.01615 (6)	0.49521 (6)	0.0585 (3)	0.0186 (4)	
H15A	0.027315	0.480095	−0.025658	0.022*	
H15B	0.041679	0.523411	0.101427	0.022*	

### N',N'''-((1E,1'E)-{[Butane-1,4-diylbis(oxy)]\ bis(2,1-phenylene)}bis(methanylylidene))bis(isonicotinohydrazide) (II) Atomic displacement parameters (Å^2^)

**Table:**

	*U*^11^	*U*^22^	*U*^33^	*U*^12^	*U*^13^	*U*^23^
O1	0.0225 (7)	0.0348 (8)	0.0211 (6)	0.0142 (6)	−0.0014 (5)	0.0027 (6)
O2	0.0183 (6)	0.0283 (7)	0.0212 (6)	0.0143 (6)	−0.0004 (5)	0.0057 (5)
N1	0.0244 (9)	0.0535 (12)	0.0251 (9)	0.0188 (9)	0.0017 (7)	−0.0010 (8)
N2	0.0211 (8)	0.0262 (9)	0.0211 (8)	0.0142 (7)	0.0002 (6)	0.0028 (7)
N3	0.0177 (8)	0.0254 (8)	0.0232 (8)	0.0132 (7)	0.0008 (6)	0.0005 (6)
C1	0.0206 (9)	0.0237 (9)	0.0228 (9)	0.0113 (8)	−0.0012 (7)	−0.0003 (7)
C2	0.0322 (12)	0.094 (2)	0.0212 (10)	0.0365 (14)	0.0023 (9)	0.0105 (12)
C3	0.0279 (12)	0.092 (2)	0.0276 (11)	0.0325 (13)	−0.0028 (9)	0.0021 (12)
C4	0.0447 (15)	0.110 (2)	0.0207 (10)	0.0492 (17)	0.0037 (10)	0.0085 (13)
C5	0.0340 (13)	0.087 (2)	0.0291 (11)	0.0399 (14)	−0.0010 (10)	0.0054 (12)
C6	0.0206 (9)	0.0199 (9)	0.0205 (9)	0.0108 (8)	−0.0020 (7)	−0.0010 (7)
C7	0.0232 (9)	0.0244 (10)	0.0186 (8)	0.0149 (8)	−0.0006 (7)	0.0017 (7)
C8	0.0202 (9)	0.0204 (9)	0.0206 (8)	0.0118 (8)	−0.0023 (7)	−0.0034 (7)
C9	0.0255 (10)	0.0283 (10)	0.0233 (9)	0.0174 (9)	−0.0037 (8)	0.0000 (8)
C10	0.0298 (10)	0.0259 (10)	0.0203 (9)	0.0162 (9)	0.0024 (8)	0.0045 (8)
C11	0.0204 (9)	0.0228 (9)	0.0223 (9)	0.0113 (8)	0.0009 (7)	−0.0027 (7)
C12	0.0178 (9)	0.0194 (9)	0.0206 (8)	0.0103 (7)	−0.0043 (7)	−0.0043 (7)
C13	0.0204 (9)	0.0169 (8)	0.0172 (8)	0.0103 (7)	−0.0024 (7)	−0.0027 (7)
C14	0.0157 (8)	0.0215 (9)	0.0193 (8)	0.0116 (7)	−0.0022 (7)	−0.0004 (7)
C15	0.0173 (9)	0.0198 (9)	0.0199 (8)	0.0102 (8)	−0.0011 (7)	−0.0003 (7)

### N',N'''-((1E,1'E)-{[Butane-1,4-diylbis(oxy)]\ bis(2,1-phenylene)}bis(methanylylidene))bis(isonicotinohydrazide) (II) Geometric parameters (Å, º)

**Table:**

O1—C6	1.223 (2)	C7—C8	1.463 (3)
O2—C13	1.368 (2)	C7—H7	0.9300
O2—C14	1.433 (2)	C8—C9	1.399 (3)
N1—C3	1.318 (3)	C8—C13	1.405 (2)
N1—C4	1.331 (3)	C9—C10	1.379 (3)
N2—C6	1.355 (2)	C9—H9	0.9300
N2—N3	1.388 (2)	C10—C11	1.391 (3)
N2—H2N	0.91 (2)	C10—H10	0.9300
N3—C7	1.278 (2)	C11—C12	1.389 (3)
C1—C2	1.371 (3)	C11—H11	0.9300
C1—C5	1.379 (3)	C12—C13	1.390 (2)
C1—C6	1.501 (3)	C12—H12	0.9300
C2—C3	1.380 (3)	C14—C15	1.513 (2)
C2—H2	0.9300	C14—H14A	0.9700
C3—H3	0.9300	C14—H14B	0.9700
C4—C5	1.381 (3)	C15—C15^i^	1.527 (3)
C4—H4	0.9300	C15—H15A	0.9700
C5—H5	0.9300	C15—H15B	0.9700
			
C13—O2—C14	118.16 (13)	C13—C8—C7	119.36 (16)
C3—N1—C4	116.33 (19)	C10—C9—C8	120.95 (17)
C6—N2—N3	118.93 (15)	C10—C9—H9	119.5
C6—N2—H2N	117.6 (15)	C8—C9—H9	119.5
N3—N2—H2N	122.5 (15)	C9—C10—C11	119.22 (17)
C7—N3—N2	115.07 (15)	C9—C10—H10	120.4
C2—C1—C5	116.64 (18)	C11—C10—H10	120.4
C2—C1—C6	118.21 (17)	C12—C11—C10	121.25 (17)
C5—C1—C6	124.87 (17)	C12—C11—H11	119.4
C1—C2—C3	120.44 (19)	C10—C11—H11	119.4
C1—C2—H2	119.8	C11—C12—C13	119.25 (17)
C3—C2—H2	119.8	C11—C12—H12	120.4
N1—C3—C2	123.3 (2)	C13—C12—H12	120.4
N1—C3—H3	118.4	O2—C13—C12	124.04 (16)
C2—C3—H3	118.4	O2—C13—C8	115.63 (15)
N1—C4—C5	124.1 (2)	C12—C13—C8	120.32 (16)
N1—C4—H4	118.0	O2—C14—C15	107.28 (13)
C5—C4—H4	118.0	O2—C14—H14A	110.3
C1—C5—C4	119.1 (2)	C15—C14—H14A	110.3
C1—C5—H5	120.4	O2—C14—H14B	110.3
C4—C5—H5	120.4	C15—C14—H14B	110.3
O1—C6—N2	124.00 (17)	H14A—C14—H14B	108.5
O1—C6—C1	120.65 (16)	C14—C15—C15^i^	111.20 (18)
N2—C6—C1	115.29 (15)	C14—C15—H15A	109.4
N3—C7—C8	121.04 (16)	C15^i^—C15—H15A	109.4
N3—C7—H7	119.5	C14—C15—H15B	109.4
C8—C7—H7	119.5	C15^i^—C15—H15B	109.4
C9—C8—C13	119.01 (17)	H15A—C15—H15B	108.0
C9—C8—C7	121.63 (16)		
			
C6—N2—N3—C7	179.08 (17)	N3—C7—C8—C13	167.81 (17)
C5—C1—C2—C3	2.7 (4)	C13—C8—C9—C10	−0.3 (3)
C6—C1—C2—C3	176.9 (2)	C7—C8—C9—C10	−179.49 (17)
C4—N1—C3—C2	−2.6 (4)	C8—C9—C10—C11	0.2 (3)
C1—C2—C3—N1	0.3 (5)	C9—C10—C11—C12	0.2 (3)
C3—N1—C4—C5	1.8 (4)	C10—C11—C12—C13	−0.5 (3)
C2—C1—C5—C4	−3.4 (4)	C14—O2—C13—C12	−7.6 (2)
C6—C1—C5—C4	−177.2 (2)	C14—O2—C13—C8	173.57 (15)
N1—C4—C5—C1	1.2 (5)	C11—C12—C13—O2	−178.45 (16)
N3—N2—C6—O1	−5.6 (3)	C11—C12—C13—C8	0.4 (3)
N3—N2—C6—C1	171.51 (15)	C9—C8—C13—O2	178.94 (16)
C2—C1—C6—O1	−4.9 (3)	C7—C8—C13—O2	−1.8 (2)
C5—C1—C6—O1	168.8 (2)	C9—C8—C13—C12	0.0 (3)
C2—C1—C6—N2	177.9 (2)	C7—C8—C13—C12	179.25 (16)
C5—C1—C6—N2	−8.4 (3)	C13—O2—C14—C15	−179.86 (14)
N2—N3—C7—C8	179.54 (15)	O2—C14—C15—C15^i^	−178.00 (17)
N3—C7—C8—C9	−13.0 (3)		

Symmetry code: (i) −*x*, −*y*+1, −*z*.

### N',N'''-((1E,1'E)-{[Butane-1,4-diylbis(oxy)]\ bis(2,1-phenylene)}bis(methanylylidene))bis(isonicotinohydrazide) (II) Hydrogen-bond geometry (Å, º)

Cg2 is the centroid of the C8–C13 benzene ring.

**Table:**

*D*—H···*A*	*D*—H	H···*A*	*D*···*A*	*D*—H···*A*
N2—H2*N*···N1^ii^	0.91 (2)	2.04 (2)	2.907 (2)	159 (2)
C3—H3···O1^iii^	0.93	2.60	3.449 (3)	153
C3—H3···N3^iii^	0.93	2.55	3.223 (3)	129
C7—H7···N1^ii^	0.93	2.63	3.372 (3)	137
C12—H12···O1^iv^	0.93	2.43	3.331 (2)	163
C15—H15*A*···*Cg*2^v^	0.97	2.91	3.748 (2)	145

Symmetry codes: (ii) *y*−1/3, −*x*+*y*+1/3, −*z*+1/3; (iii) *x*−*y*+2/3, *x*+1/3, −*z*+4/3; (iv) −*y*+1/3, *x*−*y*+2/3, *z*−1/3; (v) *x*, *y*, *z*−1.
