# Supplementary material for: The crystal structures and Hirshfeld surface analysis of N′,N′′′-((1E,1′E)-{[methyl­enebis(­oxy)]bis­(6-bromo-3,1-phenyl­ene)}bis­(methan­ylyl­idene))bis­(isonicotinohydrazide) dihydrate and N′,N′′′-((1E,1′E)-{[butane-1,4-diylbis(­oxy)]bis­(2,1-phenyl­ene)}bis­(methan­ylyl­idene))bis­(isonicotino­hydrazide) [+ solvent]
Source: Acta Crystallogr E Crystallogr Commun. 2019 Apr 18;75(Pt 5):655–61. doi: 10.1107/S2056989019005048 (PMC6505606; doi:10.1107/S2056989019005048)

Search Overview

Search:	search1
Date/Time done:	Wed Apr 10 17:21:31 2019
Database(s):	CSD version 5.40 updates (Feb 2019)<br>CSD version 5.40 (November 2018)
Restriction Info:	No refcode restrictions applied
Filters:	None
Percentage Completed:	100%
Number of Hits:	51

Single query used. Search found structures that:

match
Query 1

Query 1

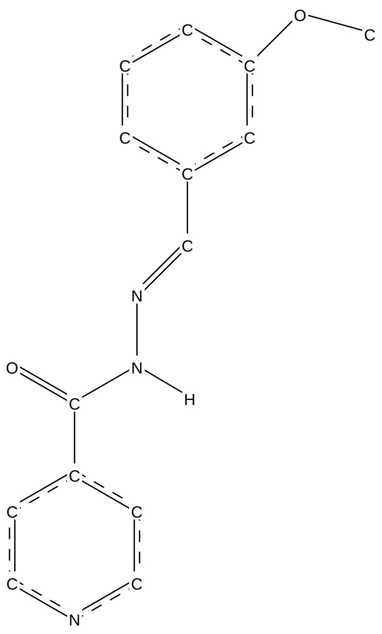

Search: search1 (Wed Apr 10 17:21:31 2019): Hits 1-4

BICFOL

Reference: S.Sivaraman, A.Agilandeswari, C.Balakrishnan, R.M.Sockalingam, S.P.Meenakshisundaram, R.Markkandan (2018) *J.Mol.Struct.* ,**1173,**385

Formula: C_{15} H_{13} N_3 O_3

Compound Name: N'-[1-(2H-1,3-benzodioxol-5-yl)ethylidene]pyridine-4-carbohydrazide

Synonym: N-(benzo[d][1,3]dioxol-5-yl)-N-methylnicotinihydrazide

Space Group:	P-1	***Cell:***	***a*** 9.080(0)	***b*** 9.114(0)	***c*** 9.939(0)
Space Group No.:	2	***(Å,°)***	α 91.06(0)	β 112.74(0)	γ 116.33(0)

R-Factor (%): 3.59 ***Temperature(K):*** 296 ***Density(g/cm³):*** 1.422

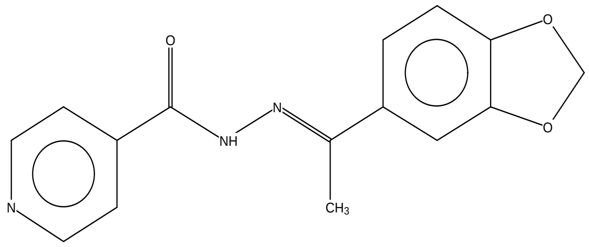

CABMEZ

Reference: Heng-Yu Qian, Zhen-Xin Zhao, Chun-Xia Zhang, Zhi-Gang Yin (2010) *Z.Kristallogr.-New Cryst.Struct.* ,**225,**383

Formula: C_{15} H_{15} N_3 O_3,C_1 H_4 O_1

Compound Name: N'-(3-Ethoxy-4-hydroxybenzylidene)isonicotinohydrazide methanol solvate

Space Group:	P21	***Cell:***	***a*** 11.279(0)	***b*** 6.443(0)	***c*** 11.890(0)
Space Group No.:	4	***(Å,°)***	α 90.00	β 109.07(0)	γ 90.00

R-Factor (%): 3.64 ***Temperature(K):*** 293 ***Density(g/cm³):*** 1.291

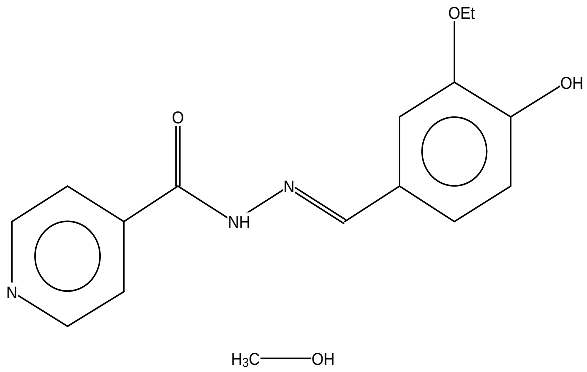

CANCOK

Reference: Ming Yu, Xin Chen, Zuo-Liang Jing (2005) *Acta Crystallogr.,Sect.E:Struct.Rep.Online* ,**61,**o1345

Formula: C_{14} H_{13} N_3 O_3

Compound Name: Isonicotinic acid (2-hydroxy-3-methoxy-benzylidene)hydrazide

Synonym: N'-(2-Hydroxy-3-methoxybenzylidene)isonicotinohydrazide

Space Group:	P21/c	***Cell:***	***a*** 7.671(2)	***b*** 16.268(5)	***c*** 10.884(3)
Space Group No.:	14	***(Å,°)***	α 90.00	β 110.42(0)	γ 90.00

R-Factor (%): 5.11 ***Temperature(K):*** 294 ***Density(g/cm³):*** 1.416

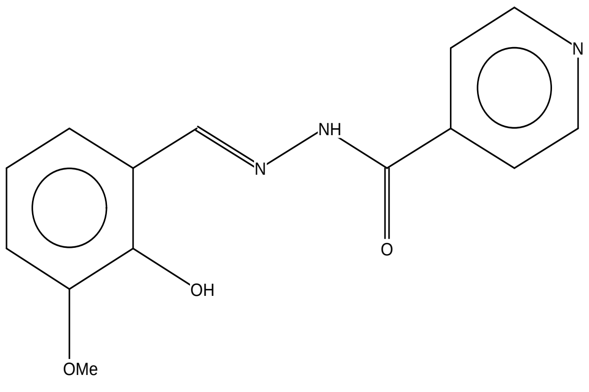

CANCOK01

Reference: De-Suo Yang (2007) *J.Chem.Cryst.* ,**37,**343

Formula: C_{14} H_{13} N_3 O_3

Compound Name: Isonicotinic acid (1-(3-methoxy-2-hydroxyphenyl)methylidene)hydrazide

Synonym: N'-(2-Hydroxy-3-methoxybenzylidene)isonicotinohydrazide

Space Group:	P21/c	***Cell:***	***a*** 7.662(1)	***b*** 16.249(2)	***c*** 10.874(2)
Space Group No.:	14	***(Å,°)***	α 90.00	β 110.43(0)	γ 90.00

R-Factor (%): 6.44 ***Temperature(K):*** 298 ***Density(g/cm³):*** 1.420

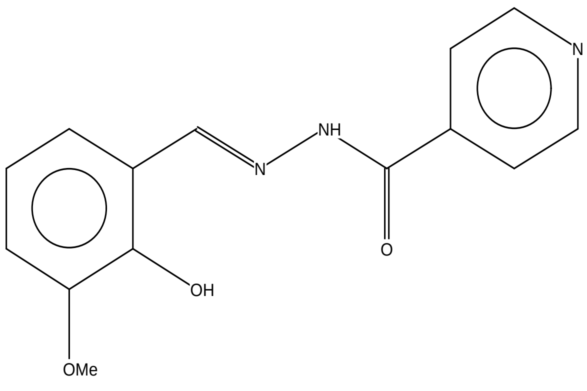

Search: search1 (Wed Apr 10 17:21:31 2019): Hits 5-8

CANCOK02

Reference: Jun Xu (2013)
Synth.React.Inorg.,Met.-Org.,Nano-Met.Chem. ,**43,**1329

Formula: $C_{14} H_{13} N_3 O_3$

Compound Name: N'-(2-Hydroxy-3-methoxybenzylidene)isonicotinohydrazide

Space Group:	P21/c	***Cell:***	***a*** 7.671(0)	***b*** 16.257(0)	***c*** 10.879(0)
Space Group No.:	14	***(Å,°)***	α 90.00	β 110.43(0)	γ 90.00
R-Factor (%):	4.11	***Temperature(K):*** 298	***Density(g/cm^3):*** 1.417		

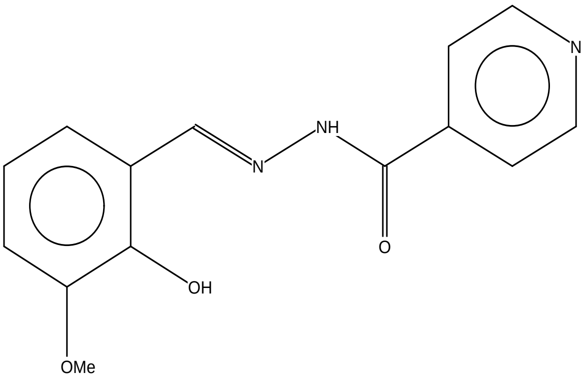

CANCOK03

Reference: M.R.J.Elsegood, M.Kose, Eyup Akgun (2015)
CSD Communication(Private Communication) ,

Formula: $C_{14} H_{13} N_3 O_3$

Compound Name: N'-(2-hydroxy-3-methoxybenzylidene)isonicotinohydrazide

Synonym: Isonicotinic acid (2-hydroxy-3-methoxy-benzylidene)hydrazide

Space Group:	P21/n	***Cell:***	***a*** 7.540(0)	***b*** 16.243(1)	***c*** 10.772(0)
Space Group No.:	14	***(Å,°)***	α 90.00	β 109.59(0)	γ 90.00
R-Factor (%):	3.90	***Temperature(K):*** 150	***Density(g/cm^3):*** 1.450		

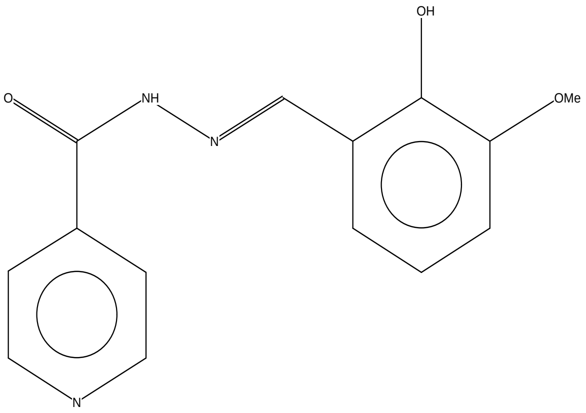

EDENUW

Reference: V.G.Bhagiratha, T.N.Chandrakantha, Puttaraja, M.K.Kokila, M.Nethaji (2000) *Mol.Mater.(Mol.Cryst.Liq.Cryst.,Sect.C)* , **12,**215

Formula: $C_{16} H_{17} N_3 O_4,2(H_2 O_1)$

Compound Name: 3,4,5-trimethoxybenzaldehyde isonicotinoyl hydrazone dihydrate

Space Group:	P21/n	***Cell:***	***a*** 9.115(4)	***b*** 16.305(3)	***c*** 11.750(5)
Space Group No.:	14	***(Å,°)***	α 90.00	β 91.43(4)	γ 90.00
R-Factor (%):	6.24	***Temperature(K):*** 295	***Density(g/cm^3):*** 1.337		

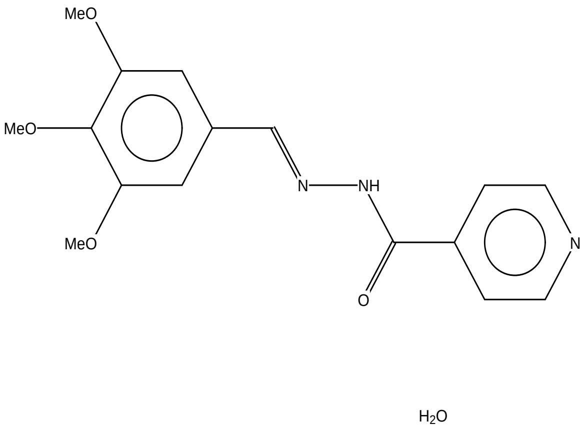

EDENUW01

Reference: M.A.Peralta, M.N.V.de Souza, S.M.S.V.Wardell, J.L.Wardell, J.N.Low, C.Glidewell (2007)
Acta Crystallogr.,Sect.C:Cryst.Struct.Commun. ,**63,**o68

Formula: $C_{16} H_{17} N_3 O_4,2(H_2 O_1)$

Compound Name: 3,4,5-Trimethoxybenzaldehyde isonicotinoylhydrazone dihydrate

Space Group:	P21/n	***Cell:***	***a*** 8.993(0)	***b*** 16.271(1)	***c*** 11.631(0)
Space Group No.:	14	***(Å,°)***	α 90.00	β 90.35(0)	γ 90.00
R-Factor (%):	6.47	***Temperature(K):*** 120	***Density(g/cm^3):*** 1.371		

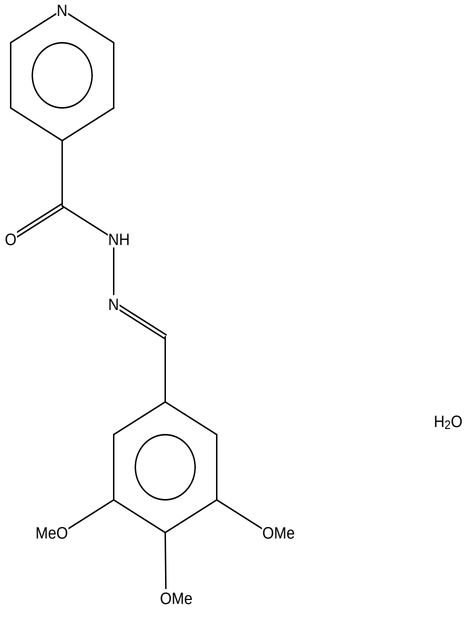

Search: search1 (Wed Apr 10 17:21:31 2019): Hits 9-12

GAWSAZ

Reference: Jun Shi (2005) *Acta Crystallogr.,Sect.E:Struct.Rep.Online* , **61,**o3933

Formula: $C_{21} H_{19} N_3 O_3$

Compound Name: (E)-N'-(4-Benzyloxy-3-methoxybenzylidene)isonicotinohydrazide

Space Group:	Pbca	***Cell:***	***a***	11.042(1)	***b***	8.071(1)	***c***	41.313(7)
Space Group No.:	61	***(Å,°)***	α	90.00	β	90.00	γ	90.00

R-Factor (%): 4.69 ***Temperature(K):*** 294 ***Density(g/cm^3):*** 1.304

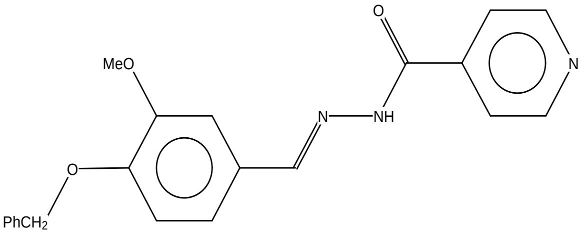

GEPFAJ

Reference: Heng-Yu Qian, Zhi-Gang Yin, Jia Jia, Sheng-Min Liu, Ling-Qin Feng (2006) *Acta Crystallogr.,Sect.E:Struct.Rep.Online* ,**62,** o3623

Formula: $C_{15} H_{15} N_3 O_3$

Compound Name: N'-(3-Ethoxy-4-hydroxybenzylidene)isonicotinohydrazide

Space Group:	P21/c	***Cell:***	***a***	13.631(3)	***b***	12.075(2)	***c***	8.777(2)
Space Group No.:	14	***(Å,°)***	α	90.00	β	96.70(3)	γ	90.00

R-Factor (%): 3.50 ***Temperature(K):*** 298 ***Density(g/cm^3):*** 1.321

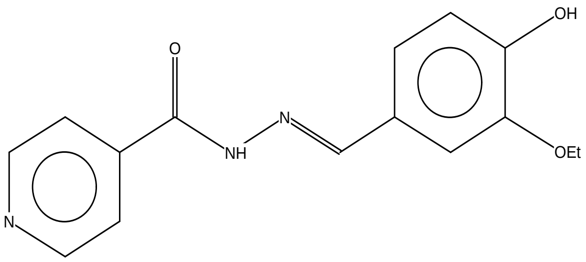

GINPID

Reference: Yi-Feng Sun, Xin-Li Wang, Ji-Kun Li, Xue-Li Cheng, Ren-Tao Wu (2007) *Acta Crystallogr.,Sect.E:Struct.Rep.Online* ,**63,** o4467

Formula: $C_{15} H_{15} N_3 O_4,H_2 O_1$

Compound Name: 4-Hydroxy-3,5-dimethoxybenzaldehyde isonicotinoylhydrazone monohydrate

Space Group:	P21/c	***Cell:***	***a***	9.014(1)	***b***	21.663(3)	***c***	7.820(1)
Space Group No.:	14	***(Å,°)***	α	90.00	β	93.65(0)	γ	90.00

R-Factor (%): 4.19 ***Temperature(K):*** 273 ***Density(g/cm^3):*** 1.392

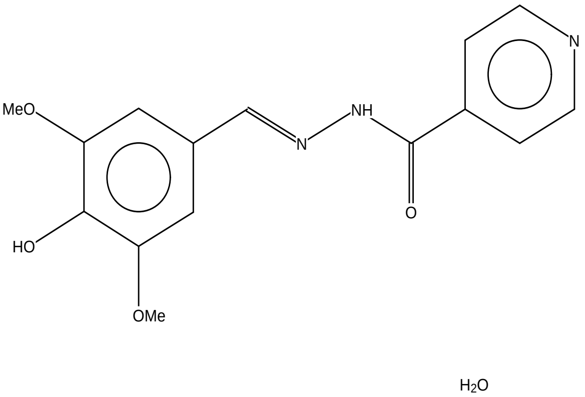

HESQOM

Reference: Qiao-Zhen Zhang, Yan-Li Zhao, Xin Chen, Ming Yu (2006) *Acta Crystallogr.,Sect.E:Struct.Rep.Online* ,**62,**o5432

Formula: $C_{22} H_{20} N_4 O_5$

Compound Name: (E)-N'-(3-Ethoxy-4-(4-nitrobenzyloxy)benzylidene)isonicotinohydrazide

Space Group:	P21/c	***Cell:***	***a***	12.454(3)	***b***	11.163(2)	***c***	14.923(3)
Space Group No.:	14	***(Å,°)***	α	90.00	β	103.82(3)	γ	90.00

R-Factor (%): 5.71 ***Temperature(K):*** 294 ***Density(g/cm^3):*** 1.386

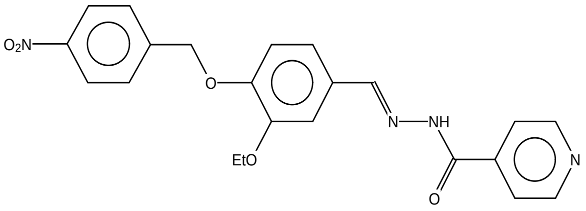

Search: search1 (Wed Apr 10 17:21:31 2019): Hits 13-16

HESRUT

Reference: Jian-Rong Han, Xiao-Li Zhen, Xia Tian, Shou-Xin Liu (2006) *Acta Crystallogr.,Sect.E:Struct.Rep.Online* ,**62,**o5570

Formula: $C_{21} H_{18} N_4 O_5$

Compound Name: (E)-N'-(3-Methoxy-2-(2-nitrobenzyloxy)benzylidene) isonicotinohydrazide

Space Group:	P21/c	***Cell:***	***a*** 7.897(1)	***b*** 26.120(6)	***c*** 20.139(4)		
Space Group No.:	14	***(Å,°)***	α 90.00	β 96.36(0)	γ 90.00		
R-Factor (%):	5.71	***Temperature(K):***	294	***Density(g/cm³):***	1.308		

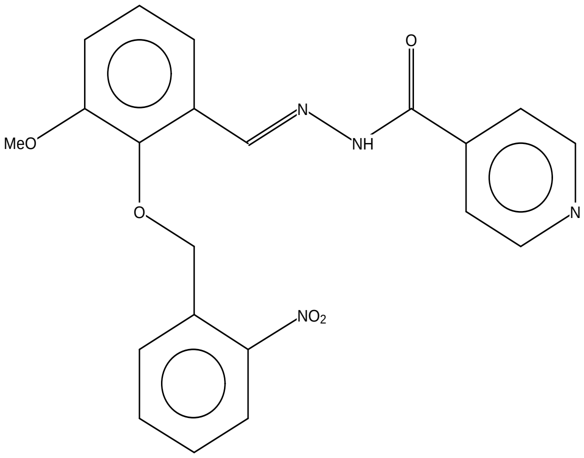

HEXJOM

Reference: V.Vrdoljak, B.Prugovecki, I.Primozic, T.Hrenar, D.Cvijanovic, J.P.Vukovic, R.Odzak, M.Skocibusic, S.Prugovecki, J.Lovric, D.Matkovic-Calogovic, M.Cindric (2018) *New J.Chem.* ,**42,** 11697

Formula: $C_{14} H_{14} N_3 O_3{}^{1+}$,$C_1 H_4 O_1$,$Br_1{}^{1-}$

Compound Name: 4-{-2-[(2-hydroxy-3-methoxyphenyl) methylidene]hydrazinecarbonyl}pyridin-1-ium bromide methanol solvate

Space Group:	P21/n	***Cell:***	***a*** 13.368(0)	***b*** 7.636(0)	***c*** 16.454(1)
Space Group No.:	14	***(Å,°)***	α 90.00	β 98.02(0)	γ 90.00
R-Factor (%):	5.46	***Temperature(K):***	293	***Density(g/cm³):***	1.534

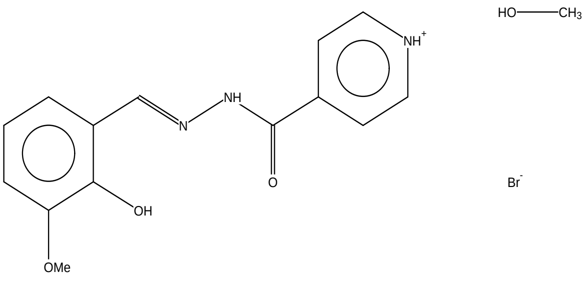

IGABEY

Reference: Xiao-Li Zhen, Xiao-Liu Li (2008) *Acta Crystallogr.,Sect.E:Struct.Rep.Online* ,**64,**o2170

Formula: $C_{20} H_{15} Cl_1 N_4 O_7 S_1$,$4(C_2 H_4 O_2)$

Compound Name: (E)-N'-(2-(4-Chloro-3-nitrophenylsulfonyloxy)-3-methoxybenzylidene) isonicotinohydrazide acetic acid solvate

Space Group:	P-1	***Cell:***	***a*** 8.056(1)	***b*** 13.876(3)	***c*** 16.097(3)
Space Group No.:	2	***(Å,°)***	α 79.01(3)	β 76.01(3)	γ 75.44(3)
R-Factor (%):	5.94	***Temperature(K):***	294	***Density(g/cm³):***	1.451

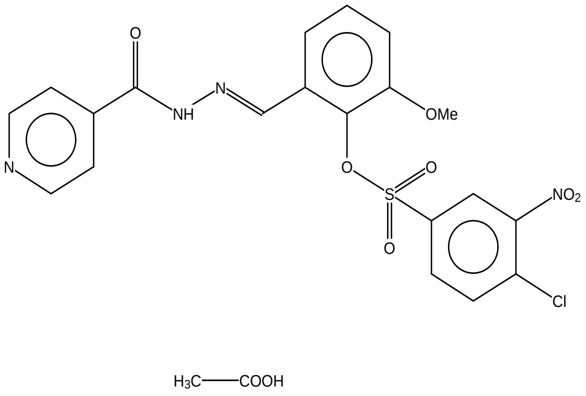

IGALIN

Reference: M.K.Prasanna, M.Sithambaresan, K.Pradeepkumar, M.R.P.Kurup (2013) *Acta Crystallogr.,Sect.E:Struct.Rep.Online* ,**69,** o881

Formula: $C_{14} H_{13} N_3 O_3$,$H_2 O_1$

Compound Name: N'-[(E)-2-Hydroxy-5-methoxybenzylidene]pyridine-4-carbohydrazide monohydrate

Synonym: N'-(2-Hydroxy-5-methoxybenzylidene)isonicotinohydrazide monohydrate

Space Group:	Pna21	***Cell:***	***a*** 12.646(1)	***b*** 12.742(1)	***c*** 8.931(0)
Space Group No.:	33	***(Å,°)***	α 90.00	β 90.00	γ 90.00
R-Factor (%):	4.54	***Temperature(K):***	296	***Density(g/cm³):***	1.335

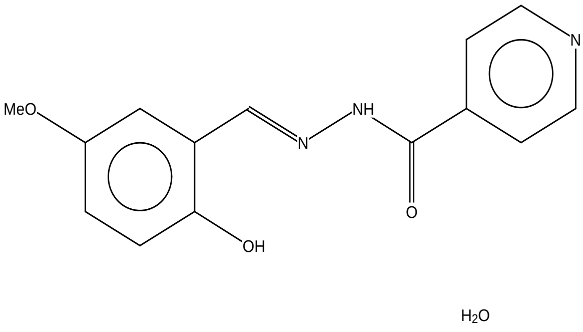

Search: search1 (Wed Apr 10 17:21:31 2019): Hits 17-20

JIRWOX

Reference: Chun-Hua Diao, Zhi Fan, Xin Chen (2007) *Acta Crystallogr.,Sect.C:Cryst.Struct.Commun.* ,**63,**o717

Formula: $C_{21}\ H_{18}\ Cl_1\ N_3\ O_5\ S_1,C_2\ H_4\ O_2$

Compound Name: (E)-N'-(5-Chloro-3-methoxy-2-(4-methylphenylsulfonyloxy)benzylidene) isonicotinohydrazide acetic acid solvate

Space Group: Pna21 ***Cell:*** ***a*** 14.886(3) ***b*** 23.190(4) ***c*** 7.064(1)
Space Group No.: 33 ***(Å,°)*** α 90.00 β 90.00 γ 90.00

R-Factor (%): 4.76 ***Temperature(K):*** 294 ***Density(g/cm3):*** 1.416

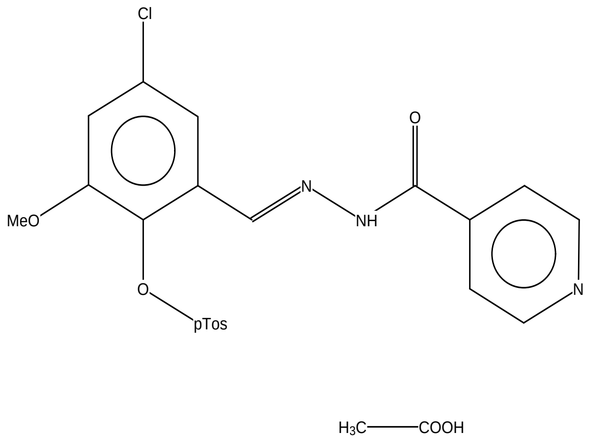

LEXPUA

Reference: Xue-Fang Shi, Lei He, Guo-Zhun Ma, Cui-Cui Yuan (2007) *Acta Crystallogr.,Sect.E:Struct.Rep.Online* ,**63,**o1119

Formula: $C_{14}\ H_{13}\ N_3\ O_3,H_2\ O_1$

Compound Name: N'-(4-Hydroxy-3-methoxybenzylidene)isonicotinohydrazide monohydrate

Space Group: P21/n ***Cell:*** ***a*** 13.343(3) ***b*** 6.528(1) ***c*** 16.005(3)
Space Group No.: 14 ***(Å,°)*** α 90.00 β 98.42(0) γ 90.00

R-Factor (%): 3.88 ***Temperature(K):*** 294 ***Density(g/cm3):*** 1.393

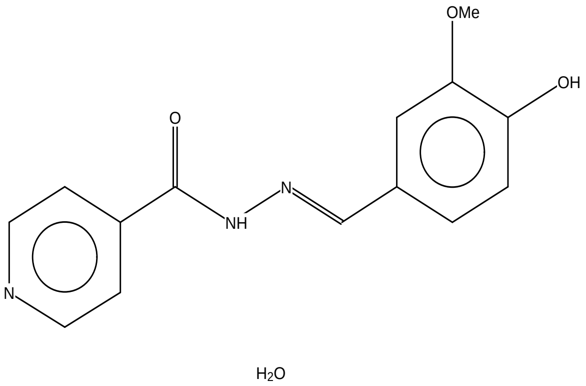

LEXPUA01

Reference: K.R.Roopashree, B.Inturi, G.V.Pujar, S.R.Prem Kumar, H.C.Devarajegowda (2016) *IUCrData* ,**1,**x161300

Formula: $C_{14}\ H_{13}\ N_3\ O_3,H_2\ O_1$

Compound Name: N'-[(1E)-4-hydroxy-3-methoxybenzylidene]isonicotinohydrazide monohydrate

Space Group: P21/n ***Cell:*** ***a*** 8.369(0) ***b*** 13.091(1) ***c*** 12.678(1)
Space Group No.: 14 ***(Å,°)*** α 90.00 β 99.09(0) γ 90.00

R-Factor (%): 4.29 ***Temperature(K):*** 296 ***Density(g/cm3):*** 1.401

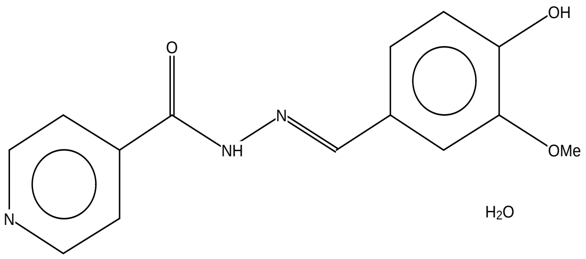

LEXPUA02

Reference: V.Ferraresi-Curotto, G.A.Echeverria, O.E.Piro, R.Pis-Diez, A.C.Gonzalez-Baro (2017) *J.Mol.Struct.* ,**1133,**436

Formula: $C_{14}\ H_{13}\ N_3\ O_3,H_2\ O_1$

Compound Name: N'-((4-hydroxy-3-methoxyphenyl)methylidene)pyridine-4-carbohydrazide monohydrate

Space Group: P21/n ***Cell:*** ***a*** 8.374(0) ***b*** 13.103(1) ***c*** 12.683(1)
Space Group No.: 14 ***(Å,°)*** α 90.00 β 99.02(1) γ 90.00

R-Factor (%): 5.78 ***Temperature(K):*** 293 ***Density(g/cm3):*** 1.398

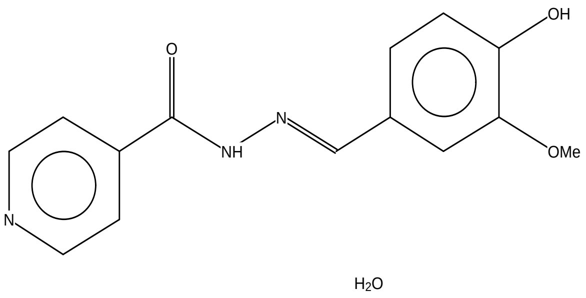

Search: search1 (Wed Apr 10 17:21:31 2019): Hits 21-24

MAZFIF

Reference:	Reza Kia, H.Kargar (2015) *J.Coord.Chem.* ,**68,**1441
Formula:	$C_{15} H_{15} N_3 O_3$
Compound Name:	N'-(3-ethoxy-2-hydroxybenzylidene)isonicotinohydrazide

Space Group:	P21/n	***Cell:***	***a*** 7.441(1)	***b*** 10.545(1)	***c*** 17.958(3)		
Space Group No.:	14	***(Å,°)***	α 90.00	β 93.32(1)	γ 90.00		
R-Factor (%):	4.07	***Temperature(K):***	296	***Density(g/cm^3):***	1.347		

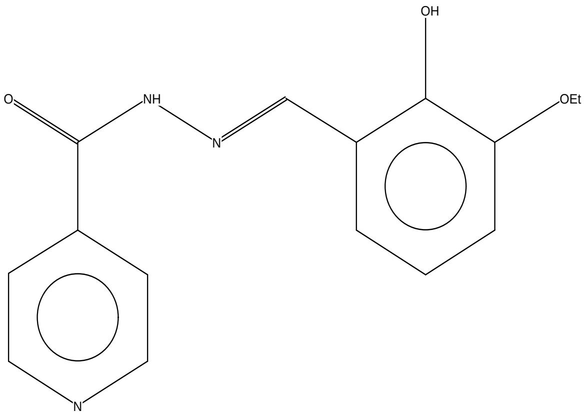

MUDGAV

Reference:	M.Tabatabaee, A.Taghinezhadkoshknou, M.Dusek, K.Fejfarova (2015) *Synth.React.Inorg.,Met.-Org.,Nano-Met.Chem.* ,**45,** 1506
Formula:	$C_{14} H_{14} N_3 O_3$ $^{1+}$,$N_1 O_3$ $^{1-}$
Compound Name:	4-([2-(2-hydroxy-3-methoxybenzylidene)hydrazinyl]carbonyl)pyridinium nitrate

Space Group:	Pna21	***Cell:***	***a*** 19.540(0)	***b*** 7.495(0)	***c*** 20.004(0)
Space Group No.:	33	***(Å,°)***	α 90.00	β 90.00	γ 90.00
R-Factor (%):	2.65	***Temperature(K):***	120	***Density(g/cm^3):***	1.516

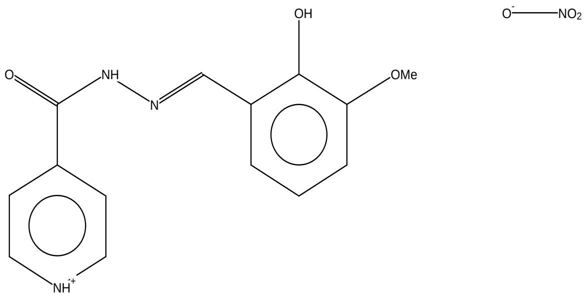

MUDGAV01

Reference:	V.Vrdoljak, B.Prugovecki, I.Primozic, T.Hrenar, D.Cvijanovic, J.P.Vukovic, R.Odzak, M.Skocibusic, S.Prugovecki, J.Lovric, D.Matkovic-Calogovic, M.Cindric (2018) *New J.Chem.* ,**42,** 11697
Formula:	$C_{14} H_{14} N_3 O_3$ $^{1+}$,$N_1 O_3$ $^{1-}$
Compound Name:	4-{2-[(2-hydroxy-3-methoxyphenyl) methylidene]hydrazinecarbonyl}pyridin-1-ium nitrate

Space Group:	P21/c	***Cell:***	***a*** 21.748(0)	***b*** 7.459(0)	***c*** 19.515(0)
Space Group No.:	14	***(Å,°)***	α 90.00	β 112.49(0)	γ 90.00
R-Factor (%):	5.32	***Temperature(K):***	293	***Density(g/cm^3):***	1.518

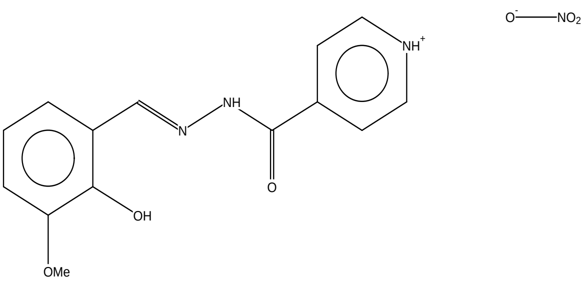

MUDGEZ

Reference:	M.Tabatabaee, A.Taghinezhadkoshknou, M.Dusek, K.Fejfarova (2015) *Synth.React.Inorg.,Met.-Org.,Nano-Met.Chem.* ,**45,** 1506
Formula:	$C_{28} H_{26} Co_1 N_6 O_6$ $^{1+}$,$N_1 O_3$ $^{1-}$,5.$_2$84($H_2 O_1$)
Compound Name:	bis(2-methoxy-6-([2-(pyridinium-4-ylcarbonyl)hydrazinylidene]methyl) phenolato)-cobalt nitrate hydrate

Space Group:	P-1	***Cell:***	***a*** 10.649(0)	***b*** 11.997(0)	***c*** 13.903(0)
Space Group No.:	2	***(Å,°)***	α 104.39(0)	β 110.15(0)	γ 95.38(0)
R-Factor (%):	5.45	***Temperature(K):***	120	***Density(g/cm^3):***	1.591

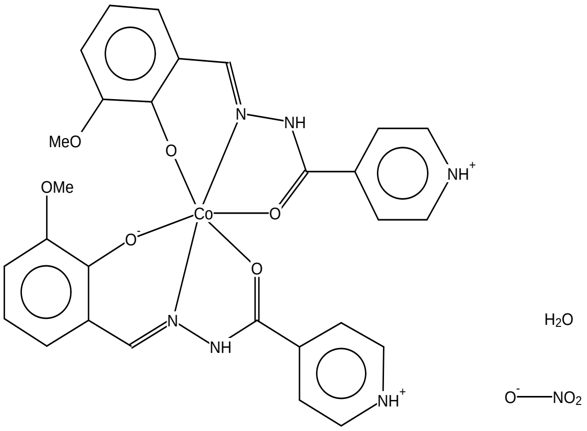

Search: search1 (Wed Apr 10 17:21:31 2019): Hits 25-28

NABNOW

Reference: Li Li, Yuan Zhuo Zhang, E Liu, Chengxiong Yang, J.A.Golen, A.L.Rheingold, Guoqi Zhang (2016) *J.Mol.Struct.* ,**1110,**180

Formula: $C_{15} H_{16} N_3 O_3$ $^{1+}$,$N_1 O_3$ $^{1-}$

Compound Name: 4-((2-(3-ethoxy-2-hydroxybenzylidene)hydrazino)carbonyl)pyridinium nitrate

Space Group:	P-1	***Cell:***	***a*** 6.753(0)	***b*** 6.967(0)	***c*** 16.554(1)
Space Group No.:	2	***(Å,°)***	α 81.19(0)	β 81.25(0)	γ 81.67(0)

R-Factor (%): 3.71 ***Temperature(K):*** 100 ***Density(g/cm^3):*** 1.533

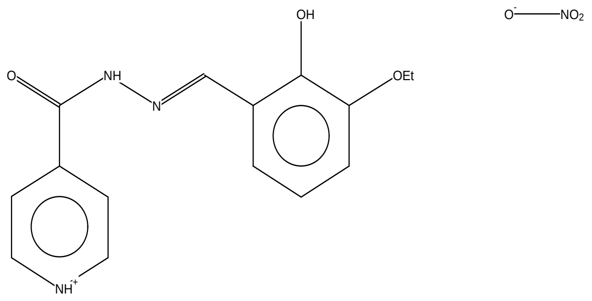

NUDXAN

Reference: Min Hong, Honglin Geng, Meiju Niu, Fei Wang, Dacheng Li, Jifeng Liu, Handong Yin (2014) *Eur.J.Med.Chem.* ,**86,**550

Formula: $C_{17} H_{21} N_3 O_3 Sn_1$

Compound Name: (N'-(3-methoxy-2-oxybenzylidene)isonicotinohydrazide)-trimethyl-tin unknown solvate

Synonym: N'-(3-methoxy-2-((trimethylstannyl)oxy)benzylidene)isonicotinohydrazide unknown solvate

Space Group:	R-3	***Cell:***	***a*** 31.643(8)	***b*** 31.643(8)	***c*** 12.083(1)
Space Group No.:	148	***(Å,°)***	α 90.00	β 90.00	γ 120.00

R-Factor (%): 7.92 ***Temperature(K):*** 293 ***Density(g/cm^3):*** 1.238

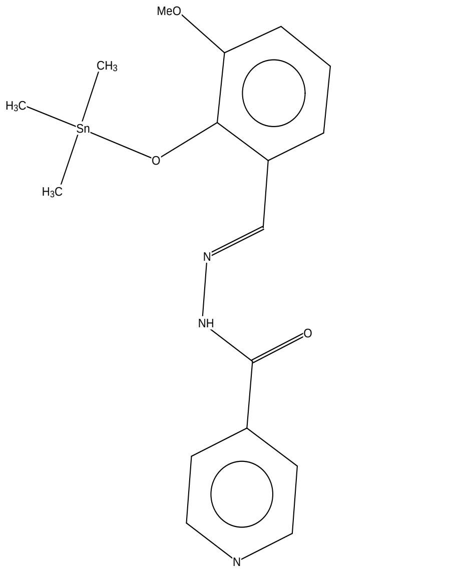

NUQZUV

Reference: H.S.Naveenkumar, A.Sadikun, P.Ibrahim, C.S.Yeap, H.-K.Fun (2010) *Acta Crystallogr.,Sect.E:Struct.Rep.Online* ,**66,**o1231

Formula: $C_{16} H_{17} N_3 O_4$

Compound Name: (E)-N'-(2,3,4-Trimethoxybenzylidene)isonicotinohydrazide

Space Group:	P21/c	***Cell:***	***a*** 14.246(3)	***b*** 9.397(2)	***c*** 12.098(3)
Space Group No.:	14	***(Å,°)***	α 90.00	β 109.25(0)	γ 90.00

R-Factor (%): 4.74 ***Temperature(K):*** 100 ***Density(g/cm^3):*** 1.370

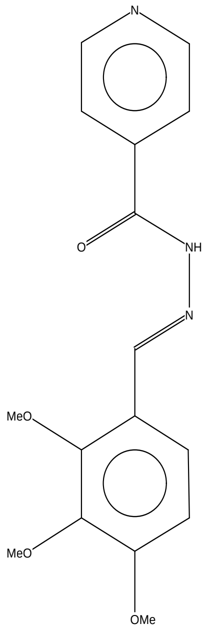

NURBEI

Reference: H.S.Naveenkumar, A.Sadikun, P.Ibrahim, C.S.Yeap, H.-K.Fun (2010) *Acta Crystallogr.,Sect.E:Struct.Rep.Online* ,**66,**o1235

Formula: $C_{16} H_{17} N_3 O_4$,2($H_2 O_1$)

Compound Name: (E)-N'-(2,4,5-Trimethoxybenzylidene)isonicotinohydrazide dihydrate

Synonym: N'-(2,4,5-Trimethoxybenzylidene)isonicotinohydrazide dihydrate

Space Group:	P21	***Cell:***	***a*** 6.816(0)	***b*** 14.565(1)	***c*** 8.559(0)
Space Group No.:	4	***(Å,°)***	α 90.00	β 103.42(0)	γ 90.00

R-Factor (%): 3.40 ***Temperature(K):*** 100 ***Density(g/cm^3):*** 1.412

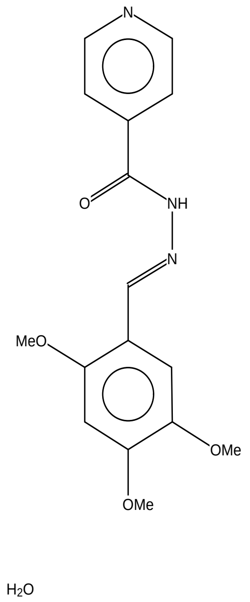

Search: search1 (Wed Apr 10 17:21:31 2019): Hits 29-32

ODAROB

Reference: Shao-Wen Chen, Han-Dong Yin, Da-Qi Wang, Xia Kong, Xiao-Fang Chen (2006) *Acta Crystallogr.,Sect.E:Struct.Rep.Online* ,**62,** o2043

Formula: $C_{14} H_{14} N_3 O_3 {}^{1+}, Cl_1 {}^{1-}, 0.5(H_2 O_1)$

Compound Name: 2-Hydroxy-3-methoxybenzaldehyde (pyridinium-4-ylcarbonyl)hydrazone chloride hemihydrate

Space Group:	P21/n	***Cell:***	***a*** 12.925(3)	***b*** 7.437(1)	***c*** 15.503(4)		
Space Group No.:	14	***(Å,°)***	α 90.00	β 90.98(0)	γ 90.00		
R-Factor (%):	5.02	***Temperature(K):*** 293	***Density(g/cm³):*** 1.412				

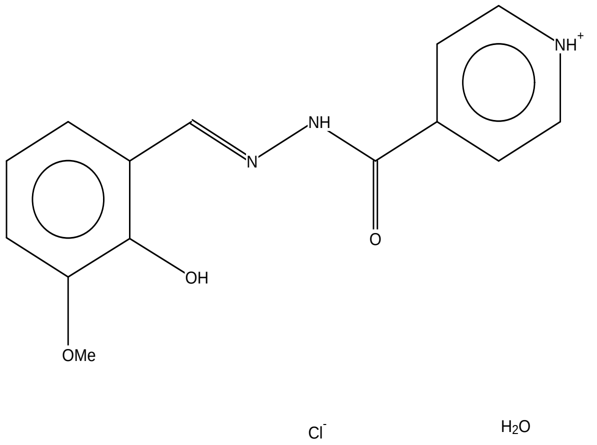

PESFOJ

Reference: Chun-Hua Diao, Ming Yu (2006) *Acta Crystallogr.,Sect.E:Struct.Rep.Online* ,**62,**o5209

Formula: $C_{21} H_{18} N_4 O_5$

Compound Name: (E)-N'-(3-Methoxy-4-(4-nitrobenzyloxy)benzylidene) isonicotinohydrazide

Space Group:	Pccn	***Cell:***	***a*** 23.601(5)	***b*** 10.798(2)	***c*** 14.698(3)		
Space Group No.:	56	***(Å,°)***	α 90.00	β 90.00	γ 90.00		
R-Factor (%):	5.40	***Temperature(K):*** 294	***Density(g/cm³):*** 1.441				

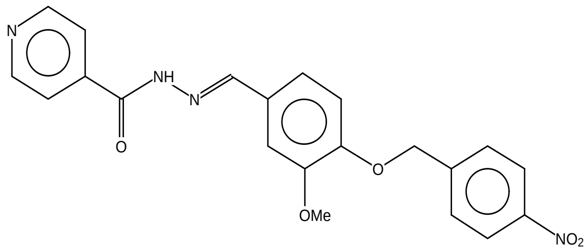

PILHUO

Reference: Jian-Rong Han, Hui-Jun Wang, Xiao-Li Zhen, Xia Tian (2007) *Acta Crystallogr.,Sect.E:Struct.Rep.Online* ,**63,**o4013

Formula: $C_{21} H_{16} Cl_1 N_3 O_4$

Compound Name: (E)-N'-(4-(4-Chlorobenzoyloxy)-3-methoxybenzylidene) isonicotinohydrazide

Space Group:	P21/c	***Cell:***	***a*** 21.746(6)	***b*** 12.011(3)	***c*** 7.700(2)		
Space Group No.:	14	***(Å,°)***	α 90.00	β 99.47(0)	γ 90.00		
R-Factor (%):	4.29	***Temperature(K):*** 294	***Density(g/cm³):*** 1.372				

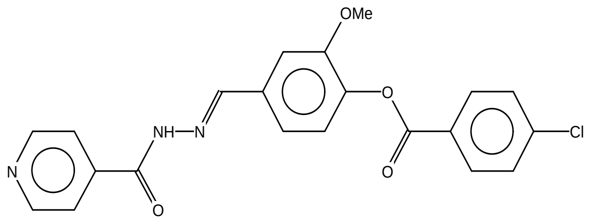

PILNAA

Reference: Zhi-gang Yin, Heng-yu Qian, Feng Yu-li, Chen Yu-zhen (2007) *Acta Crystallogr.,Sect.E:Struct.Rep.Online* ,**63,**o4109

Formula: $C_{14} H_{11} N_3 O_3$

Compound Name: (E)-N'-(1,3-Benzodioxol-5-ylmethylene)isonicotinohydrazide

Space Group:	P-1	***Cell:***	***a*** 7.919(1)	***b*** 10.955(2)	***c*** 15.479(3)		
Space Group No.:	2	***(Å,°)***	α 78.88(3)	β 80.11(3)	γ 69.08(3)		
R-Factor (%):	8.30	***Temperature(K):*** 298	***Density(g/cm³):*** 1.462				

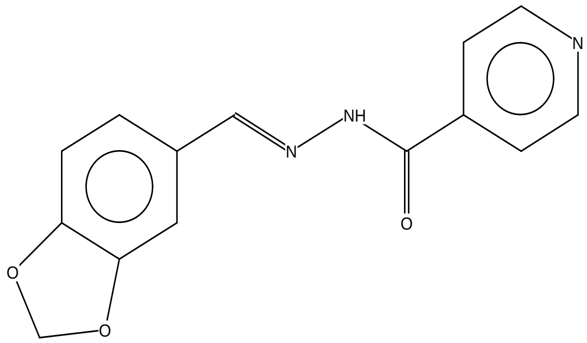

Search: search1 (Wed Apr 10 17:21:31 2019): Hits 33-36

PUFNEK

Reference: H.S.Naveenkumar, A.Sadikun, P.Ibrahim, W.-S.Loh, H.-K.Fun (2009) *Acta Crystallogr.,Sect.E:Struct.Rep.Online* ,**65,**o2540

Formula: $C_{21} H_{19} N_3 O_3$

Compound Name: (E)-N'-(3-Benzyloxy-4-methoxybenzylidene)isonicotinohydrazide

Space Group:	P21/c	***Cell:***	***a*** 18.393(0)	***b*** 11.557(0)	***c*** 8.351(0)		
Space Group No.:	14	***(Å,°)***	α 90.00	β 93.44(0)	γ 90.00		
R-Factor (%):	6.00	***Temperature(K):***	100	***Density(g/cm³):***	1.355		

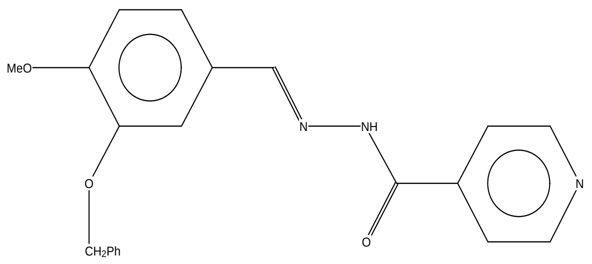

PUHCEB

Reference: Z.Shafiq, M.Yaqub, M.N.Tahir, A.Hussain, M.S.Iqbal (2009) *Acta Crystallogr.,Sect.E:Struct.Rep.Online* ,**65,**o2899

Formula: $C_{14} H_{13} N_3 O_3$

Compound Name: N'-[(E)-4-Hydroxy-3-methoxybenzylidene]pyridine-4-carbohydrazide

Synonym: N'-(4-Hydroxy-3-methoxybenzylidene)isonicotinohydrazide

Space Group:	Cc	***Cell:***	***a*** 14.854(1)	***b*** 12.494(0)	***c*** 7.716(0)
Space Group No.:	9	***(Å,°)***	α 90.00	β 116.72(0)	γ 90.00
R-Factor (%):	3.35	***Temperature(K):***	296	***Density(g/cm³):***	1.409

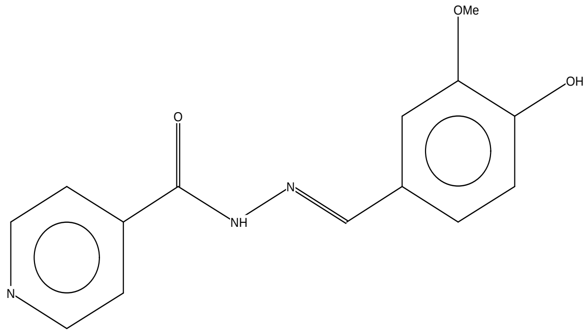

RAVRUB

Reference: Wei Chen, X.Zhang, B.Shan, X.You (1997) *Acta Crystallogr.,Sect.C:Cryst.Struct.Commun.* ,**53,**775

Formula: $C_{15} H_{15} N_3 O_3$

Compound Name: 3,4-Dimethoxybenzaldehyde isonicotinoylhydrazone

Space Group:	P21/c	***Cell:***	***a*** 11.810(1)	***b*** 13.804(0)	***c*** 8.925(1)
Space Group No.:	14	***(Å,°)***	α 90.00	β 101.32(0)	γ 90.00
R-Factor (%):	5.95	***Temperature(K):***	295	***Density(g/cm³):***	1.328

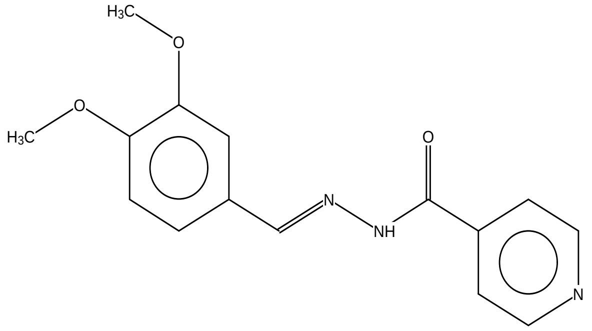

RIHQEG

Reference: H.Hosseini-Monfared, R.Bikas, Joaquin Sanchiz, Tadeusz Lis, M.Siczek, Jiri Tucek, R.Zboril, P.Mayer (2013) *Polyhedron* ,**61,**45

Formula: $C_{32} H_{38} Cl_2 Mn_1 N_6 O_8$

Compound Name: trans-dichloro-bis(N'-((2-hydroxy-3-methoxyphenyl)methylene) isonicotinohydrazide)-bis(ethanol)-manganese(ii)

Space Group:	P-1	***Cell:***	***a*** 8.222(3)	***b*** 8.918(3)	***c*** 12.458(4)
Space Group No.:	2	***(Å,°)***	α 69.33(5)	β 89.78(5)	γ 74.71(5)
R-Factor (%):	4.56	***Temperature(K):***	100	***Density(g/cm³):***	1.539

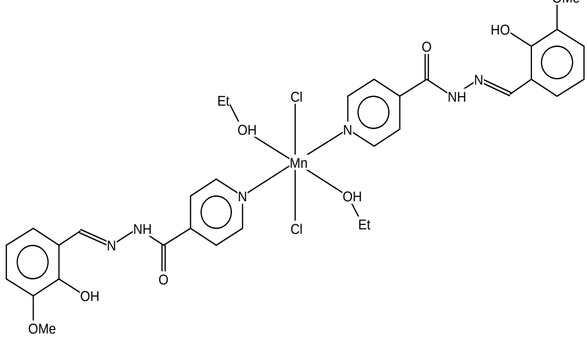

Search: search1 (Wed Apr 10 17:21:31 2019): Hits 37-40

RISHIL

Reference: Xia Liu, Xue-Fang Shi (2007) *Acta Crystallogr.,Sect.E:Struct.Rep.Online* ,**63,**o4807

Formula: $C_{14} H_{13} N_3 O_3, C_1 H_4 O_1$

Compound Name: N'-(4-Hydroxy-3-methoxybenzylidene)isonicotinohydrazide methanol solvate

Space Group:	P21/c	***Cell:***	***a***	13.274(1)	***b***	6.379(0)	***c***	17.538(1)
Space Group No.:	14	***(Å,°)***	α	90.00	β	105.15(0)	γ	90.00

R-Factor (%): 4.45 ***Temperature(K):*** 113 ***Density(g/cm^3):*** 1.406

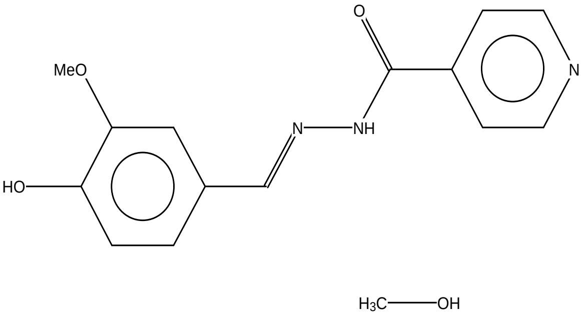

ROFXIU

Reference: San-Jun Peng, Hai-Yun Hou (2008) *Acta Crystallogr.,Sect.E:Struct.Rep.Online* ,**64,**o1995

Formula: $C_{14} H_{12} Br_1 N_3 O_3$

Compound Name: N'-(5-Bromo-2-hydroxy-3-methoxybenzylidene)isonicotinohydrazide

Space Group:	P21/c	***Cell:***	***a***	7.494(0)	***b***	15.884(1)	***c***	11.799(1)
Space Group No.:	14	***(Å,°)***	α	90.00	β	99.78(0)	γ	90.00

R-Factor (%): 3.04 ***Temperature(K):*** 298 ***Density(g/cm^3):*** 1.680

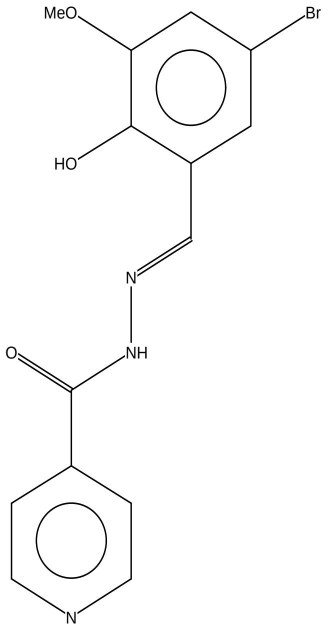

URIKAJ

Reference: E.Loukopoulos, K.Griffiths, G.R.Akien, N.Kourkoumelis, A.Abdul-Sada, G.E.Kostakis (2015) *Inorganics* ,**3,**448

Formula: $(C_{42} H_{36} Cl_2 Dy_2 N_9 O_9\ ^{1+})n,n(Cl_1\ ^{1-}),n(C_1 H_4 O_1),6n(H_2 O_1)$

Compound Name: catena-[tris(μ-N'-(2-oxy-3-methoxybenzylidene)isonicotinohydrazide)-dichloro-di-dysprosium chloride methanol solvate hexahydrate]

Space Group:	P21/n	***Cell:***	***a***	14.287(0)	***b***	19.984(0)	***c***	20.064(0)
Space Group No.:	14	***(Å,°)***	α	90.00	β	101.86(0)	γ	90.00

R-Factor (%): 4.52 ***Temperature(K):*** 173 ***Density(g/cm^3):*** 1.638

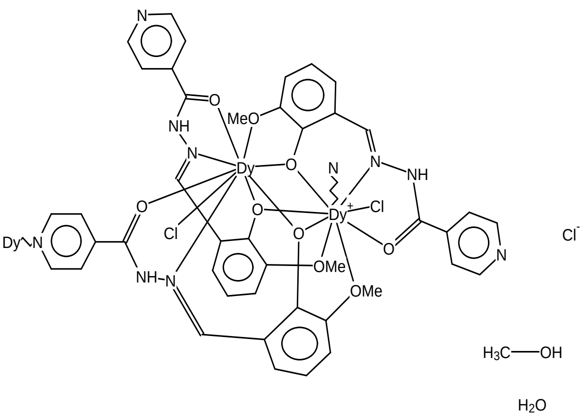

URIKEN

Reference: E.Loukopoulos, K.Griffiths, G.R.Akien, N.Kourkoumelis, A.Abdul-Sada, G.E.Kostakis (2015) *Inorganics* ,**3,**448

Formula: $(C_{42} H_{36} Cl_2 Gd_2 N_9 O_9\ ^{1+})n,n(Cl_1\ ^{1-}),6n(H_2 O_1)$

Compound Name: catena-[tris(μ-N'-(2-oxy-3-methoxybenzylidene)isonicotinohydrazide)-dichloro-di-gadolinium chloride hexahydrate]

Space Group:	P21/n	***Cell:***	***a***	14.218(3)	***b***	20.386(4)	***c***	20.455(4)
Space Group No.:	14	***(Å,°)***	α	90.00	β	101.98(1)	γ	90.00

R-Factor (%): 6.08 ***Temperature(K):*** 173 ***Density(g/cm^3):*** 1.534

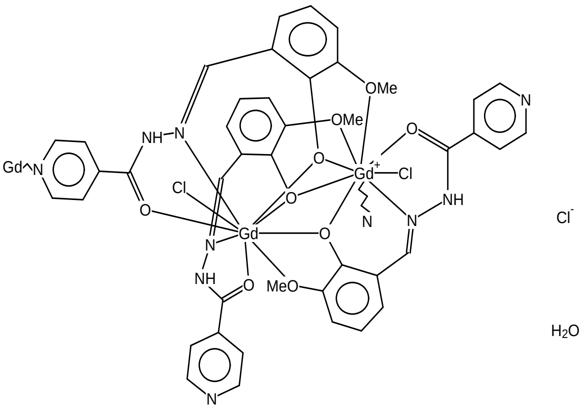

Search: search1 (Wed Apr 10 17:21:31 2019): Hits 41-44

URIKIR

Reference: E.Loukopoulos, K.Griffiths, G.R.Akien, N.Kourkoumelis, A.Abdul-Sada, G.E.Kostakis (2015) *Inorganics* ,**3**,448

Formula: C_{16} H_{20} Dy_1 N_5 O_{11}

Compound Name: bis(nitrato)-(N'-(2-oxy-3-methoxybenzylidene)isonicotinohydrazido)-bis(methanol)-dysprosium

Space Group:	P-1	***Cell:***	***a*** 10.342(0)	***b*** 14.585(0)	***c*** 15.731(0)
Space Group No.:	2	***(Å,°)***	α 95.67(0)	β 95.38(0)	γ 109.77(0)

R-Factor (%): 3.10 ***Temperature(K):*** 173 ***Density(g/cm³):*** 1.873

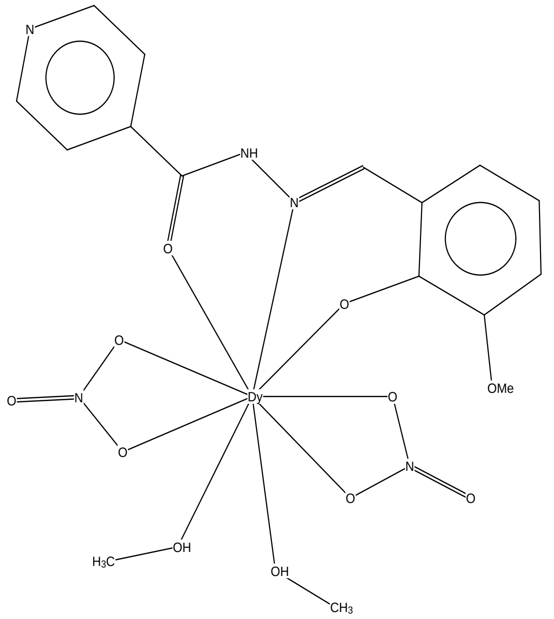

URIKOX

Reference: E.Loukopoulos, K.Griffiths, G.R.Akien, N.Kourkoumelis, A.Abdul-Sada, G.E.Kostakis (2015) *Inorganics* ,**3**,448

Formula: C_{30} H_{32} N_6 O_8 Y_1 $^{1+}$,C_2 H_3 N_1,Cl_1 $^{1-}$

Compound Name: bis(N'-(2-oxy-3-methoxybenzylidene)isonicotinohydrazido)-bis(methanol)-yttrium(iii) chloride acetonitrile solvate

Space Group:	P21/c	***Cell:***	***a*** 12.290(0)	***b*** 15.467(1)	***c*** 18.778(1)
Space Group No.:	14	***(Å,°)***	α 90.00	β 100.09(0)	γ 90.00

R-Factor (%): 5.24 ***Temperature(K):*** 100 ***Density(g/cm³):*** 1.455

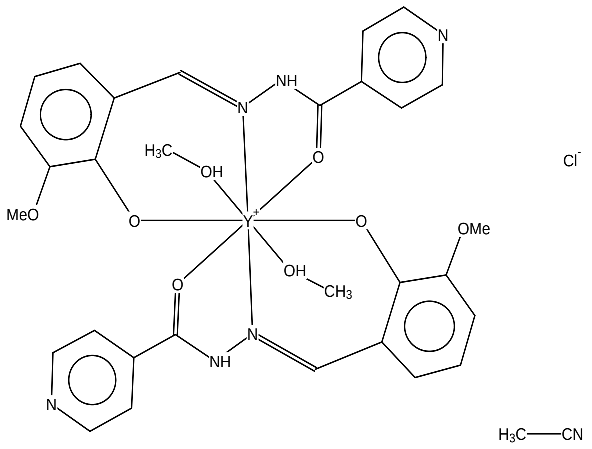

VACHAK

Reference: H.Kargar, R.Kia, M.Akkurt, O.Buyukgungor (2010) *Acta Crystallogr.,Sect.E:Struct.Rep.Online* ,**66,**o2982

Formula: C_{14} H_{13} N_3 O_3

Compound Name: N'-[(E)-2-Hydroxy-5-methoxybenzylidene]pyridine-4-carbohydrazide

Synonym: N'-(2-Hydroxy-5-methoxybenzylidene)isonicotinohydrazide

Space Group:	Cc	***Cell:***	***a*** 6.111(0)	***b*** 29.489(3)	***c*** 7.482(0)
Space Group No.:	9	***(Å,°)***	α 90.00	β 96.70(0)	γ 90.00

R-Factor (%): 4.61 ***Temperature(K):*** 296 ***Density(g/cm³):*** 1.345

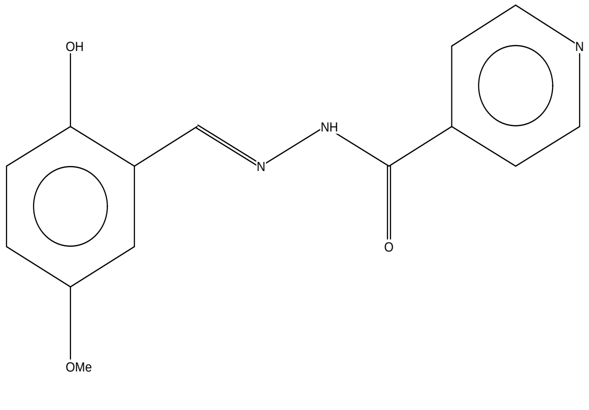

VAKBOA

Reference: Po-Heng Lin, S.Gorelsky, D.Savard, T.J.Burchell, W.Wernsdorfer, R.Clerac, M.Murugesu (2010) *Dalton Trans.* ,**39,**7650

Formula: C_{30} H_{34} Cl_2 Mn_1 N_6 O_8

Compound Name: Dichloro-bis(N'-(2-hydroxy-3-methoxybenzylidene)isonicotinohydrazide-N)-bis(methanol)-manganese(ii)

Synonym: Dichloro-bis(N'-((2-hydroxy-3-methoxyphenyl)methylidene)pyridine-4-carbohydrazide-N)-bis(methanol)-manganese(ii)

Space Group:	P-1	***Cell:***	***a*** 7.887(1)	***b*** 8.980(1)	***c*** 12.543(2)
Space Group No.:	2	***(Å,°)***	α 69.36(0)	β 76.85(0)	γ 77.74(0)

R-Factor (%): 4.08 ***Temperature(K):*** 202 ***Density(g/cm³):*** 1.519

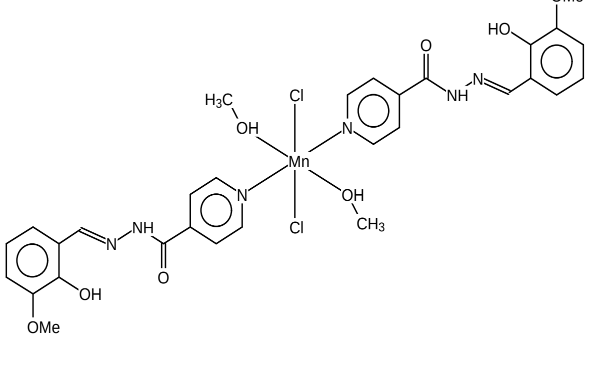

Search: search1 (Wed Apr 10 17:21:31 2019): Hits 45-48

WEVXOL

Reference: M.A.Peralta, M.N.V.de Souza, S.M.S.V.Wardell, J.L.Wardell, J.N.Low, C.Glidewell (2007) *Acta Crystallogr.,Sect.C:Cryst.Struct.Commun.* ,**63,**o68

Formula: $C_{15} H_{15} N_3 O_3,C_1 H_1 Cl_3$

Compound Name: 2,3-Dimethoxybenzaldehyde isonicotinoylhydrazone chloroform solvate

Space Group: P21/n ***Cell:*** ***a*** 12.740(0) ***b*** 10.860(0) ***c*** 13.919(0)
Space Group No.: 14 ***(Å,°)*** α 90.00 β 110.73(0) γ 90.00

R-Factor (%): 4.09 ***Temperature(K):*** 120 ***Density(g/cm³):*** 1.492

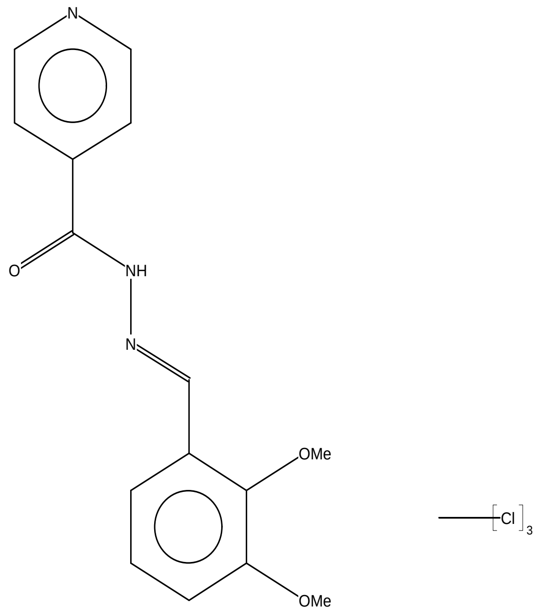

WEVXUR

Reference: M.A.Peralta, M.N.V.de Souza, S.M.S.V.Wardell, J.L.Wardell, J.N.Low, C.Glidewell (2007) *Acta Crystallogr.,Sect.C:Cryst.Struct.Commun.* ,**63,**o68

Formula: $C_{16} H_{17} N_3 O_4,H_2 O_1$

Compound Name: 3,4,5-trimethoxybenzaldehyde isonicotinoylhydrazone monohydrate

Space Group: P21/c ***Cell:*** ***a*** 10.808(0) ***b*** 10.360(0) ***c*** 14.427(0)
Space Group No.: 14 ***(Å,°)*** α 90.00 β 95.28(0) γ 90.00

R-Factor (%): 4.71 ***Temperature(K):*** 120 ***Density(g/cm³):*** 1.377

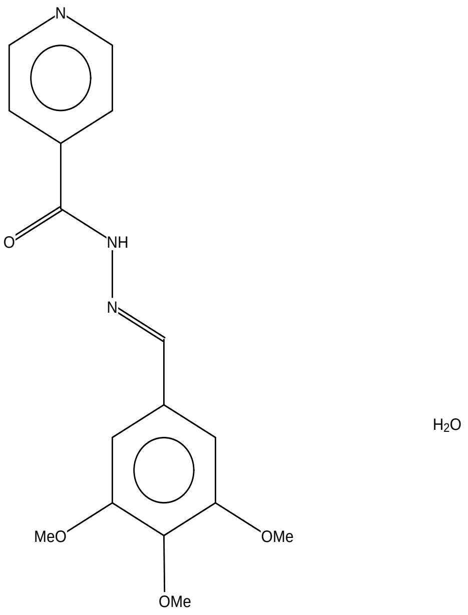

WISKEQ

Reference: Jun Xu (2013) *Synth.React.Inorg.,Met.-Org.,Nano-Met.Chem.* ,**43,**1329

Formula: $C_{28} H_{28} Cl_2 Mn_2 N_6 O_8,2(C_1 H_4 O_1)$

Compound Name: bis(μ_2-N'-(2-oxido-3-(methoxy)benzylidene)isonicotinohydrazido)-diaqua-dichloro-di-manganese(ii) methanol solvate

Space Group: P21/n ***Cell:*** ***a*** 9.498(0) ***b*** 12.136(1) ***c*** 15.341(1)
Space Group No.: 14 ***(Å,°)*** α 90.00 β 96.27(0) γ 90.00

R-Factor (%): 2.64 ***Temperature(K):*** 298 ***Density(g/cm³):*** 1.552

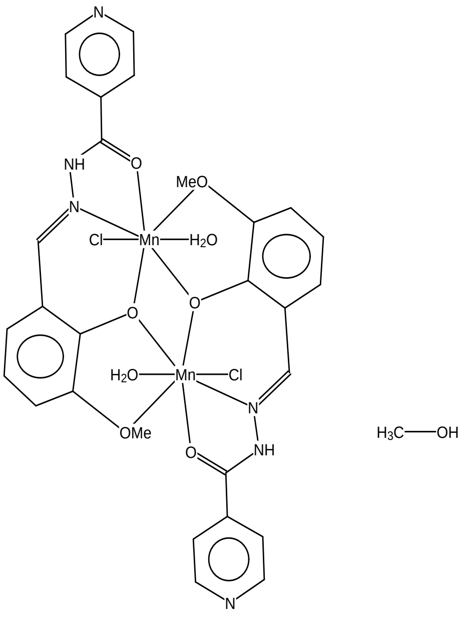

YIQFIO

Reference: S.M.S.V.Wardell, M.V.N.de Souza, J.L.Wardell, J.N.Low, C.Glidewell (2007) *Acta Crystallogr.,Sect.B:Struct.Sci.* ,**63,**879

Formula: $C_{14} H_{13} N_3 O_2$

Compound Name: (E)-N-isonicotinoyl 3-methoxybenzaldehydehydrazone

Space Group: P21/n ***Cell:*** ***a*** 8.294(0) ***b*** 11.077(0) ***c*** 13.846(5)
Space Group No.: 14 ***(Å,°)*** α 90.00 β 103.48(0) γ 90.00

R-Factor (%): 4.74 ***Temperature(K):*** 120 ***Density(g/cm³):*** 1.371

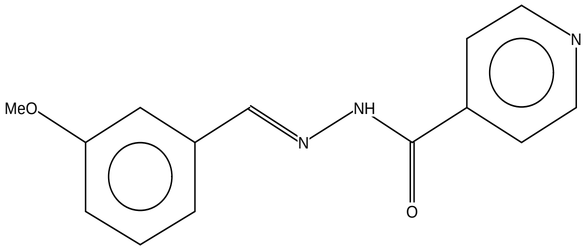

Search: search1 (Wed Apr 10 17:21:31 2019): Hits 49-51

YIQFUA

Reference: S.M.S.V.Wardell, M.V.N.de Souza, J.L.Wardell, J.N.Low, C.Glidewell (2007) *Acta Crystallogr.,Sect.B:Struct.Sci.* ,**63,**879

Formula: $C_{15} H_{15} N_3 O_2$

Compound Name: N-isonicotinoyl 3-ethoxybenzaldehydehydrazone

Space Group: P21/c	***Cell:***	***a*** 24.206(1)	***b*** 5.564(0)	***c*** 9.957(0)
Space Group No.: 14	***(Å,°)***	α 90.00	β 94.97(0)	γ 90.00

R-Factor (%): 6.86 ***Temperature(K):*** 120 ***Density(g/cm^3):*** 1.339

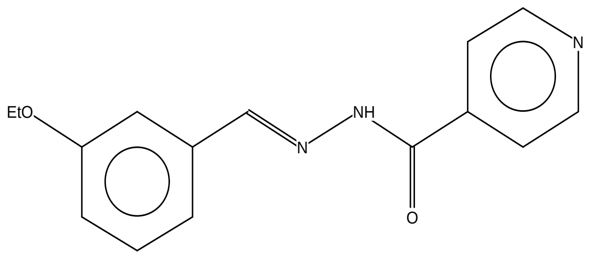

ZARHIL

Reference: Chun-Hua Diao, Zhi Fan (2012) *Acta Crystallogr.,Sect.E:Struct.Rep.Online* ,**68,**o1517

Formula: $C_{20} H_{14} Cl_1 N_3 O_3,C_2 H_4 O_2,H_2 O_1$

Compound Name: (E)-N'-[3-(4-Chlorobenzoyloxy)benzylidene]pyridine-4-carbohydrazide acetic acid solvate monohydrate

Synonym: 3-(Isonicotinoylcarbohydrazonoyl)phenyl 4-chlorobenzoate acetic acid monohydrate

Space Group: P-1	***Cell:***	***a*** 6.667(1)	***b*** 7.544(1)	***c*** 24.781(6)
Space Group No.: 2	***(Å,°)***	α 81.53(0)	β 82.97(0)	γ 66.63(0)

R-Factor (%): 4.30 ***Temperature(K):*** 294 ***Density(g/cm^3):*** 1.347

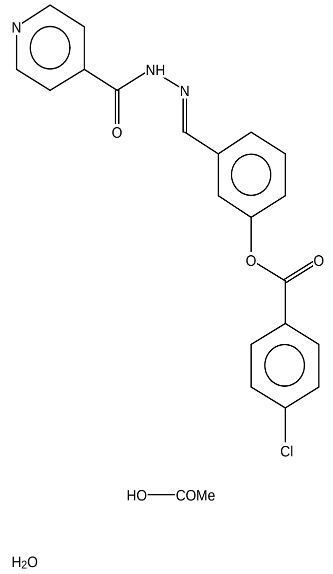

ZUVXAR

Reference: A.O.Surov, A.P.Voronin, A.A.Simagina, A.V.Churakov, Sophia Y.Skachilova, G.L.Perlovich (2015) *New J.Chem.* ,**39,**8614

Formula: $C_{14} H_{14} N_3 O_3{}^{1+},C_7 H_4 N_1 O_3 S_1{}^{1-}$

Compound Name: Isoniazid saccharinate

Space Group: P21/n	***Cell:***	***a*** 6.190(0)	***b*** 26.105(4)	***c*** 12.402(1)
Space Group No.: 14	***(Å,°)***	α 90.00	β 100.46(0)	γ 90.00

R-Factor (%): 4.63 ***Temperature(K):*** 173 ***Density(g/cm^3):*** 1.532

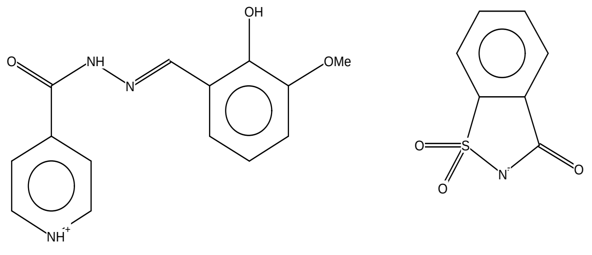

Supplement: Supplementary file 6 [file e-75-00655-sup6.pdf]
