# Supplementary material for: The crystal structures and Hirshfeld surface analysis of N′,N′′′-((1E,1′E)-{[methyl­enebis(­oxy)]bis­(6-bromo-3,1-phenyl­ene)}bis­(methan­ylyl­idene))bis­(isonicotinohydrazide) dihydrate and N′,N′′′-((1E,1′E)-{[butane-1,4-diylbis(­oxy)]bis­(2,1-phenyl­ene)}bis­(methan­ylyl­idene))bis­(isonicotino­hydrazide) [+ solvent]
Source: Acta Crystallogr E Crystallogr Commun. 2019 Apr 18;75(Pt 5):655–61. doi: 10.1107/S2056989019005048 (PMC6505606; doi:10.1107/S2056989019005048)

Search Overview

Search:	search3
Date/Time done:	Wed Apr 10 17:32:20 2019
Database(s):	CSD version 5.40 updates (Feb 2019)<br>CSD version 5.40 (November 2018)
Restriction Info:	No refcode restrictions applied
Filters:	None
Percentage Completed:	100%
Number of Hits:	23

Single query used. Search found structures that:

match

Query 1

Query 1

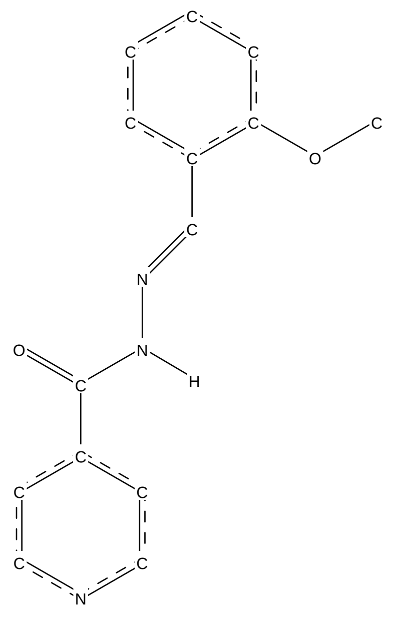

Search: search3 (Wed Apr 10 17:32:20 2019): Hits 1-4

BAXKUJ

Reference: G.Mahmoudi, Ennio Zangrando, A.Bauza, W.Maniukiewicz, R.Carballo, A.V.Gurbanov, A.Frontera (2017) *CrystEngComm* ,**19,**3322

Formula: $C_{56} H_{48} Hg_2 I_4 N_{12} O_8, 4(C_1 H_4 O_1)$

Compound Name: bis(μ-{N',N''-[ethane-1,2-diylbis(oxy-2,1-phenylenemethylylidene)]bis(pyridine-4-carbohydrazide)})-tetrakis(iodo)-di-mercury methanol solvate

Space Group:	P-1	***Cell:***	***a***	11.064(1)	***b***	12.065(1)	***c***	14.522(2)
Space Group No.:	2	***(Å,°)***	α	72.33(0)	β	69.22(0)	γ	84.58(0)

R-Factor (%): 5.25 ***Temperature(K):*** 193 ***Density(g/cm^3):*** 1.975

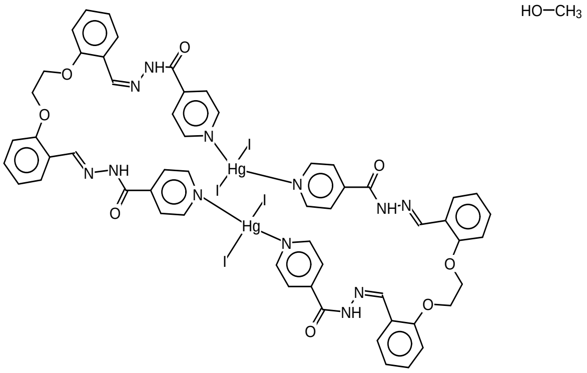

BAXLAQ

Reference: G.Mahmoudi, Ennio Zangrando, A.Bauza, W.Maniukiewicz, R.Carballo, A.V.Gurbanov, A.Frontera (2017) *CrystEngComm* ,**19,**3322

Formula: $C_{28} H_{24} N_6 O_4, 2(C_1 H_4 O_1)$

Compound Name: N',N''-[ethane-1,2-diylbis(oxy-2,1-phenylenemethylylidene)]bis(pyridine-4-carbohydrazide) methanol solvate

Space Group:	P21	***Cell:***	***a***	8.682(1)	***b***	20.009(3)	***c***	8.946(1)
Space Group No.:	4	***(Å,°)***	α	90.00	β	107.12(0)	γ	90.00

R-Factor (%): 4.30 ***Temperature(K):*** 193 ***Density(g/cm^3):*** 1.280

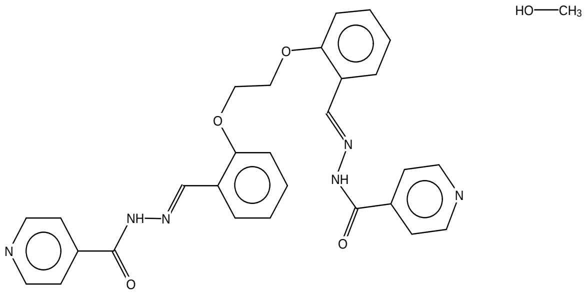

EROTEL

Reference: Hong-Yan Ban (2011) *Acta Crystallogr.,Sect.E:Struct.Rep.Online* ,**67,**o1443

Formula: $C_{14} H_{12} Br_1 N_3 O_2$

Compound Name: (E)-N'-(5-Bromo-2-methoxybenzylidene)isonicotinohydrazide

Space Group:	P21/c	***Cell:***	***a***	10.020(3)	***b***	25.732(2)	***c***	11.243(2)
Space Group No.:	14	***(Å,°)***	α	90.00	β	102.20(0)	γ	90.00

R-Factor (%): 6.37 ***Temperature(K):*** 298 ***Density(g/cm^3):*** 1.567

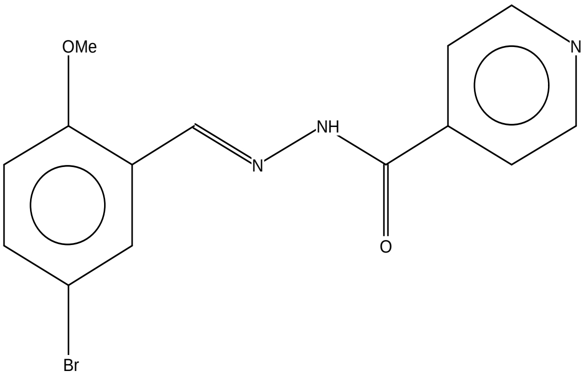

FIXJIG

Reference: X.-S.Tai, L.-H.Wang, Y.-Z.Li, M.-Y.Tan (2004) *Z.Kristallogr.-New Cryst.Struct.* ,**219,**407

Formula: $C_{28} H_{24} N_6 O_4$

Compound Name: 1,4-bis(2'-Formylphenyl)-1,4-dioxabutane bis(isonicotinoylhydrazone)

Space Group:	P-1	***Cell:***	***a***	9.512(1)	***b***	11.671(1)	***c***	12.531(1)
Space Group No.:	2	***(Å,°)***	α	106.68(3)	β	96.69(3)	γ	104.28(2)

R-Factor (%): 4.70 ***Temperature(K):*** 293 ***Density(g/cm^3):*** 1.335

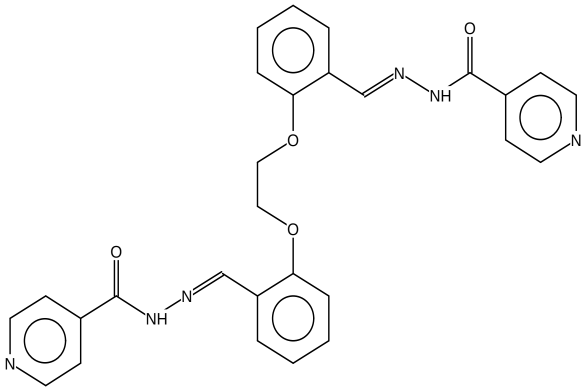

Search: search3 (Wed Apr 10 17:32:20 2019): Hits 5-8

HESRUT

Reference: Jian-Rong Han, Xiao-Li Zhen, Xia Tian, Shou-Xin Liu (2006) *Acta Crystallogr.,Sect.E:Struct.Rep.Online* ,**62,**o5570

Formula: $C_{21} H_{18} N_4 O_5$

Compound Name: (E)-N'-(3-Methoxy-2-(2-nitrobenzyloxy)benzylidene) isonicotinohydrazide

Space Group:	P21/c	***Cell:***	***a*** 7.897(1)	***b*** 26.120(6)	***c*** 20.139(4)		
Space Group No.:	14	***(Å,°)***	α 90.00	β 96.36(0)	γ 90.00		
R-Factor (%):	5.71	***Temperature(K):***	294	***Density(g/cm³):***	1.308		

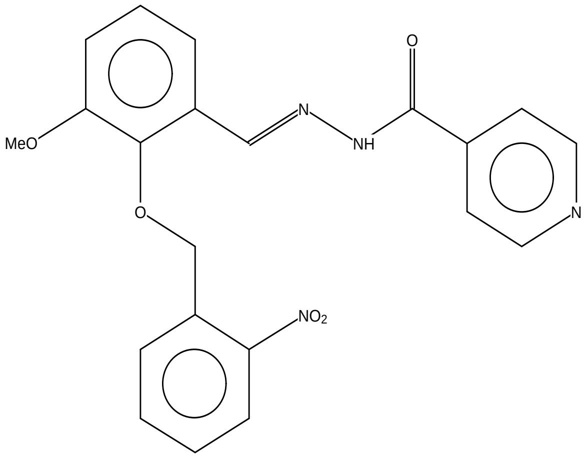

IDIWOI

Reference: Xiao-Yang Qiu, Hua-Jie Xu, Wei-Sheng Liu, Hai-Liang Zhu (2006) *Acta Crystallogr.,Sect.E:Struct.Rep.Online* ,**62,**o2304

Formula: $C_{14} H_{13} N_3 O_2$,2($H_2 O_1$)

Compound Name: (E)-N'-(2-Methoxybenzylidene)isonicotinohydrazide dihydrate

Space Group:	P-1	***Cell:***	***a*** 7.528(0)	***b*** 9.693(0)	***c*** 10.398(0)
Space Group No.:	2	***(Å,°)***	α 92.31(0)	β 100.34(0)	γ 107.93(0)
R-Factor (%):	4.62	***Temperature(K):***	298	***Density(g/cm³):***	1.369

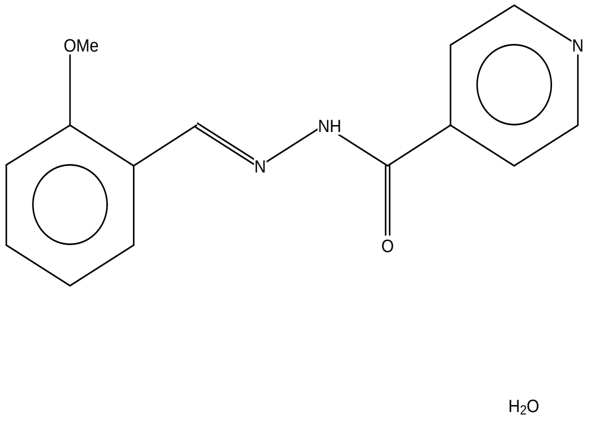

LEFQUK

Reference: S.K.Mohamed, M.A.Farrukh, M.Akkurt, M.R.Albayati, A.A.Abdelhamid (2012) *Acta Crystallogr.,Sect.E:Struct.Rep.Online* ,**68,** o2442

Formula: $C_{14} H_{13} N_3 O_2$,$C_{10} H_7 N_1 O_4$

Compound Name: (1,3-Dioxo-1,3-dihydro-2H-isoindol-2-yl)acetic acid N'-(2-methoxybenzylidene)isonicotinohydrazide

Synonym: 2-(1,3-Dioxoisoindolin-2-yl)aceticacid N'-[(E)-2-methoxybenzylidene]pyridine-4-carbohydrazide

Space Group:	P21/n	***Cell:***	***a*** 7.075(1)	***b*** 43.511(6)	***c*** 7.548(0)
Space Group No.:	14	***(Å,°)***	α 90.00	β 110.02(0)	γ 90.00
R-Factor (%):	5.52	***Temperature(K):***	296	***Density(g/cm³):***	1.401

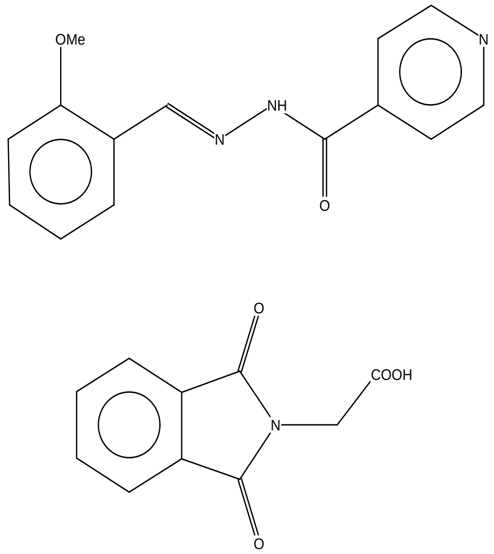

LIGXEG

Reference: Ming Dong, Ya-Wen Wang, Ai-Jiang Zhang, Yu Peng (2013) *Chem.Asian J.* ,**8,**1321

Formula: $C_{21} H_{27} N_4 O_3{}^{1+}$,$C_6 H_2 N_3 O_7{}^{1-}$

Compound Name: 4-((2-(2-(2-(Di-isopropylamino)-2-oxoethoxy)benzylidene)hydrazinyl) carbonyl)pyridinium 2,4,6-trinitrophenolate

Space Group:	Pna21	***Cell:***	***a*** 23.601(2)	***b*** 19.545(1)	***c*** 6.363(0)
Space Group No.:	33	***(Å,°)***	α 90.00	β 90.00	γ 90.00
R-Factor (%):	5.20	***Temperature(K):***	273	***Density(g/cm³):***	1.384

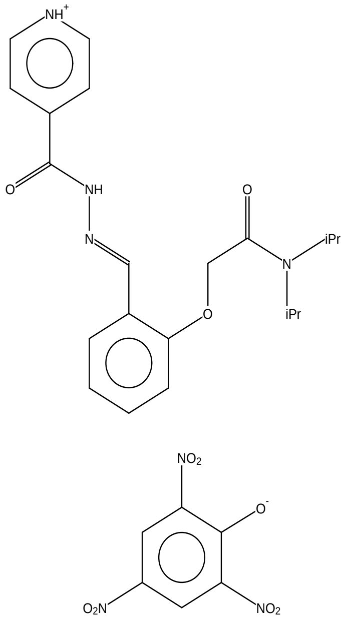

Search: search3 (Wed Apr 10 17:32:20 2019): Hits 9-12

MIQXOA

Reference: Shaokang Gao, Zhongwu Fu, Zengyou Wang (2007) *Acta Crystallogr.,Sect.E:Struct.Rep.Online* ,**63,**m3004

Formula: $C_{28} H_{34} N_6 O_8 Zn_1^{2+},2(N_1 O_3^{1-})$

Compound Name: tetra-aqua-bis(2-methoxybenzaldehyde isonicotinoylhydrazone)-zinc(ii) dinitrate

Space Group:	P21/n	***Cell:***	***a*** 7.970(4)	***b*** 17.647(9)	***c*** 11.838(4)
Space Group No.:	14	***(Å,°)***	α 90.00	β 99.06(1)	γ 90.00

R-Factor (%): 4.13 ***Temperature(K):*** 293 ***Density(g/cm^3):*** 1.559

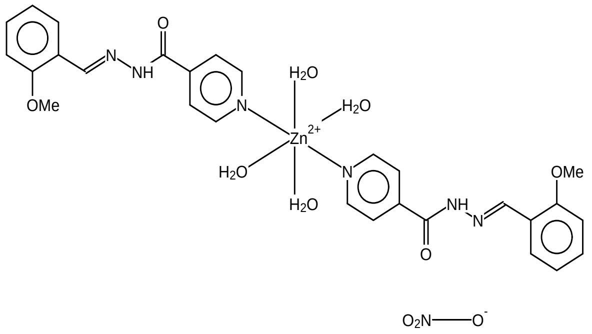

NUQZUV

Reference: H.S.Naveenkumar, A.Sadikun, P.Ibrahim, C.S.Yeap, H.-K.Fun (2010) *Acta Crystallogr.,Sect.E:Struct.Rep.Online* ,**66,**o1231

Formula: $C_{16} H_{17} N_3 O_4$

Compound Name: (E)-N'-(2,3,4-Trimethoxybenzylidene)isonicotinohydrazide

Space Group:	P21/c	***Cell:***	***a*** 14.246(3)	***b*** 9.397(2)	***c*** 12.098(3)
Space Group No.:	14	***(Å,°)***	α 90.00	β 109.25(0)	γ 90.00

R-Factor (%): 4.74 ***Temperature(K):*** 100 ***Density(g/cm^3):*** 1.370

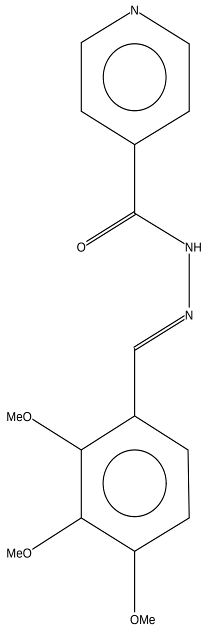

NURBEI

Reference: H.S.Naveenkumar, A.Sadikun, P.Ibrahim, C.S.Yeap, H.-K.Fun (2010) *Acta Crystallogr.,Sect.E:Struct.Rep.Online* ,**66,**o1235

Formula: $C_{16} H_{17} N_3 O_4,2(H_2 O_1)$

Compound Name: (E)-N'-(2,4,5-Trimethoxybenzylidene)isonicotinohydrazide dihydrate

Synonym: N'-(2,4,5-Trimethoxybenzylidene)isonicotinohydrazide dihydrate

Space Group:	P21	***Cell:***	***a*** 6.816(0)	***b*** 14.565(1)	***c*** 8.559(0)
Space Group No.:	4	***(Å,°)***	α 90.00	β 103.42(0)	γ 90.00

R-Factor (%): 3.40 ***Temperature(K):*** 100 ***Density(g/cm^3):*** 1.412

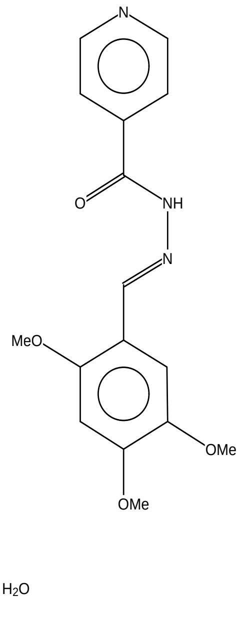

QERLIJ

Reference: Qiao-Zhen Zhang, Yan-Li Zhao, Xin Chen, Ming Yu (2006) *Acta Crystallogr.,Sect.E:Struct.Rep.Online* ,**62,**o4835

Formula: $C_{20} H_{16} Cl_1 N_3 O_2$

Compound Name: (E)-N'-(2-(4-Chlorobenzyloxy)benzylidene)isonicotinohydrazide

Space Group:	Pbca	***Cell:***	***a*** 10.212(2)	***b*** 9.696(2)	***c*** 36.927(8)
Space Group No.:	61	***(Å,°)***	α 90.00	β 90.00	γ 90.00

R-Factor (%): 4.67 ***Temperature(K):*** 294 ***Density(g/cm^3):*** 1.329

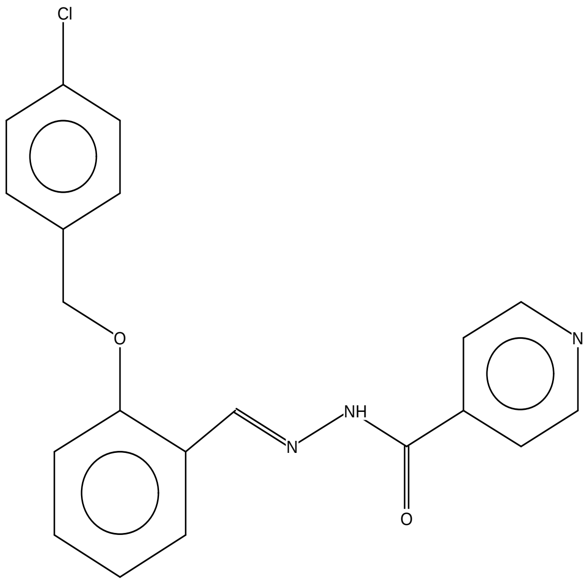

Search: search3 (Wed Apr 10 17:32:20 2019): Hits 13-16

SAZBIF

Reference: Song Yang, Shu-Ping Zhang, Ying-Ying Wu, Si-Chang Shao (2006) *Acta Crystallogr.,Sect.E:Struct.Rep.Online* ,**62,** o28

Formula: $C_{14} H_{13} N_3 O_2$

Compound Name: 2-Methoxybenzaldehyde isonicotinoylhydrazone

Space Group: P21/c ***Cell:*** ***a*** 9.766(2) ***b*** 15.935(4) ***c*** 17.428(4)
Space Group No.: 14 ***(Å,°)*** α 90.00 β 95.40(0) γ 90.00

R-Factor (%): 14.43 ***Temperature(K):*** 298 ***Density(g/cm³):*** 1.256

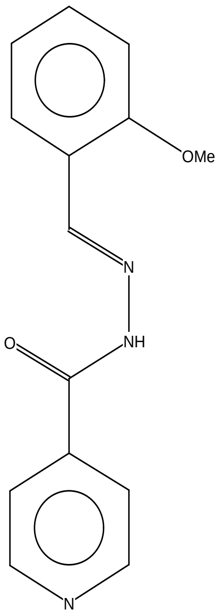

SAZBIF01

Reference: S.M.S.V.Wardell, M.V.N.de Souza, J.L.Wardell, J.N.Low, C.Glidewell (2007) *Acta Crystallogr.,Sect.B:Struct.Sci.* ,**63,**879

Formula: $C_{14} H_{13} N_3 O_2$

Compound Name: (E)-N-isonicotinoyl 2-methoxybenzaldehydehydrazone

Space Group: P21/c ***Cell:*** ***a*** 9.890(0) ***b*** 15.819(0) ***c*** 17.038(0)
Space Group No.: 14 ***(Å,°)*** α 90.00 β 97.24(0) γ 90.00

R-Factor (%): 5.23 ***Temperature(K):*** 120 ***Density(g/cm³):*** 1.282

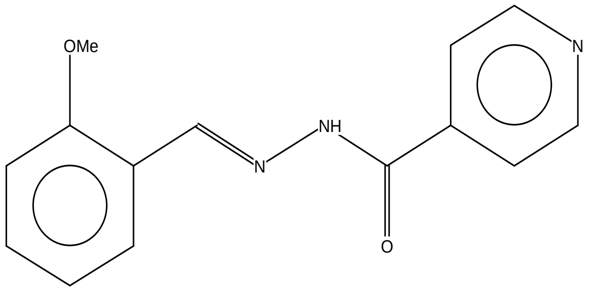

TEJQUV

Reference: Aihua Nie, S.Ghosh, Ziwei Huang (2006) *Acta Crystallogr.,Sect.E:Struct.Rep.Online* ,**62,**o1824

Formula: $C_{21} H_{16} F_1 N_3 O_2$

Compound Name: 2'-(2-(4-Fluorophenyl)chroman-4-ylidene)isonicotinohydrazide

Space Group: P21/c ***Cell:*** ***a*** 11.654(5) ***b*** 5.471(2) ***c*** 26.609(10)
Space Group No.: 14 ***(Å,°)*** α 90.00 β 102.42(0) γ 90.00

R-Factor (%): 5.10 ***Temperature(K):*** 208 ***Density(g/cm³):*** 1.449

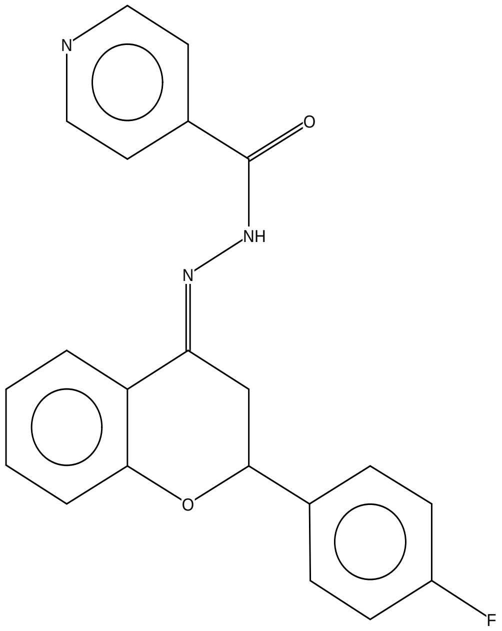

TEJQUV01

Reference: Aihua Nie, Ziwei Huang (2006) *J.Comb.Chem.* ,**8,**655

Formula: $C_{21} H_{16} F_1 N_3 O_2$

Compound Name: 2'-(2-(4-Fluorophenyl)chroman-4-ylidene)isonicotinohydrazide

Space Group: P21/c ***Cell:*** ***a*** 11.654(5) ***b*** 5.471(2) ***c*** 26.609(10)
Space Group No.: 14 ***(Å,°)*** α 90.00 β 102.42(0) γ 90.00

R-Factor (%): 5.10 ***Temperature(K):*** 208 ***Density(g/cm³):*** 1.449

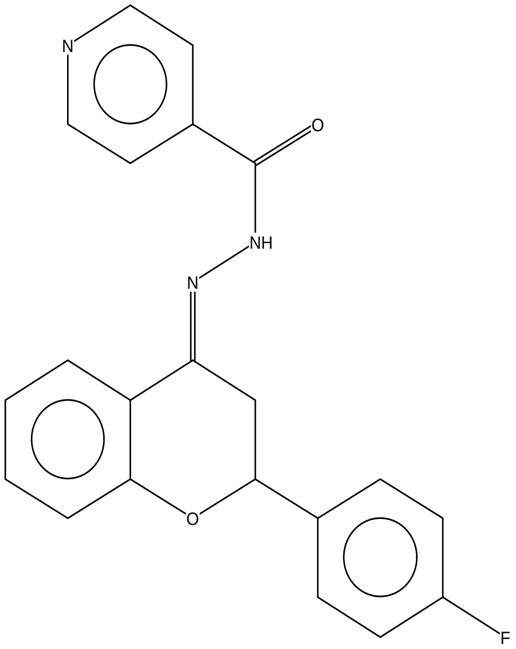

Search: search3 (Wed Apr 10 17:32:20 2019): Hits 17-20

TUPDAK

Reference: H.S.Naveenkumar, A.Sadikun, P.Ibrahim, M.Hemamalini, H.-K.Fun (2010) *Acta Crystallogr.,Sect.E:Struct.Rep.Online* ,**66,**o1337

Formula: $C_{20} H_{17} N_3 O_2, C_1 H_4 O_1, H_2 O_1$

Compound Name: (E)-N'-(2-Benzyloxybenzylidene)isonicotinohydrazide methanol solvate monohydrate

Space Group:	P21/c	***Cell:***	***a*** 17.763(3)	***b*** 12.389(1)	***c*** 8.745(1)		
Space Group No.:	14	***(Å,°)***	α 90.00	β 98.67(0)	γ 90.00		
R-Factor (%):	5.86	***Temperature(K):***	100	***Density(g/cm³):***	1.332		

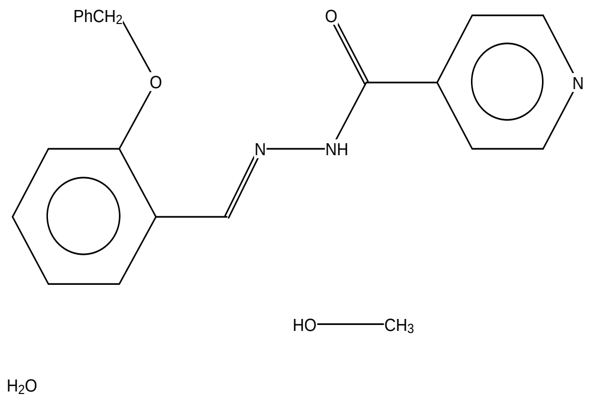

TUPDOY

Reference: Jin Hong Xia, Bao Yu Liu, Zheng Liu (2010) *Acta Crystallogr.,Sect.E:Struct.Rep.Online* ,**66,**o1341

Formula: $C_{15} H_{13} N_3 O_4$

Compound Name: 2-(2-([2-(4-Pyridylcarbonyl)hydrazinylidene]methyl)phenoxy)acetic acid

Space Group:	Pca21	***Cell:***	***a*** 12.810(1)	***b*** 4.944(0)	***c*** 21.921(2)
Space Group No.:	29	***(Å,°)***	α 90.00	β 90.00	γ 90.00
R-Factor (%):	3.26	***Temperature(K):***	296	***Density(g/cm³):***	1.432

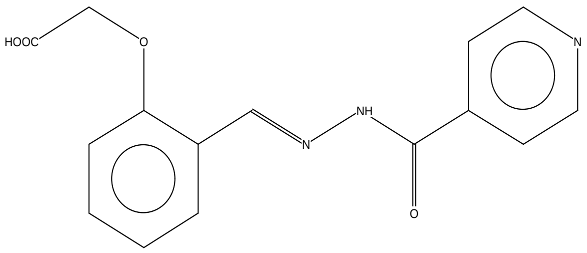

VAZTEY

Reference: Zhen-Hai Fu, Ya-Wen Wang, Yu Peng (2017) *Chem.Commun.* ,**53,**10524

Formula: $C_{27} H_{17} N_3 O_6, C_2 H_6 O_1 S_1$

Compound Name: N'-((3',6'-dihydroxy-3-oxo-3H-spiro[2-benzofuran-1,9'-xanthen]-4'-yl) methylidene)pyridine-4-carbohydrazide dimethyl sulfoxide solvate

Space Group:	P-1	***Cell:***	***a*** 7.760(1)	***b*** 13.126(4)	***c*** 14.358(3)
Space Group No.:	2	***(Å,°)***	α 106.59(2)	β 94.66(1)	γ 105.99(2)
R-Factor (%):	9.41	***Temperature(K):***	293	***Density(g/cm³):***	1.396

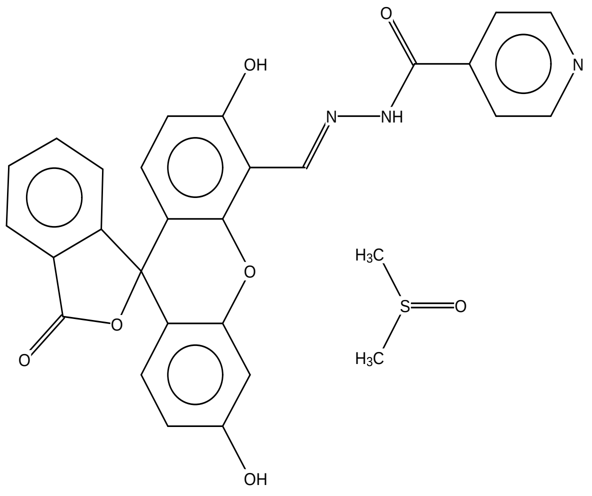

VAZTIC

Reference: Zhen-Hai Fu, Ya-Wen Wang, Yu Peng (2017) *Chem.Commun.* ,**53,**10524

Formula: $C_{34} H_{22} N_6 O_7, 2(C_2 H_6 O_1 S_1)$

Compound Name: N',N''-((3',6'-dihydroxy-3-oxo-3H-spiro[2-benzofuran-1,9'-xanthene]-4',5'-diyl)dimethylylidene)bis(pyridine-4-carbohydrazide) dimethyl sulfoxide solvate

Space Group:	P-1	***Cell:***	***a*** 8.296(0)	***b*** 15.185(1)	***c*** 16.490(1)
Space Group No.:	2	***(Å,°)***	α 83.14(0)	β 81.04(0)	γ 87.48(0)
R-Factor (%):	8.11	***Temperature(K):***	293	***Density(g/cm³):***	1.277

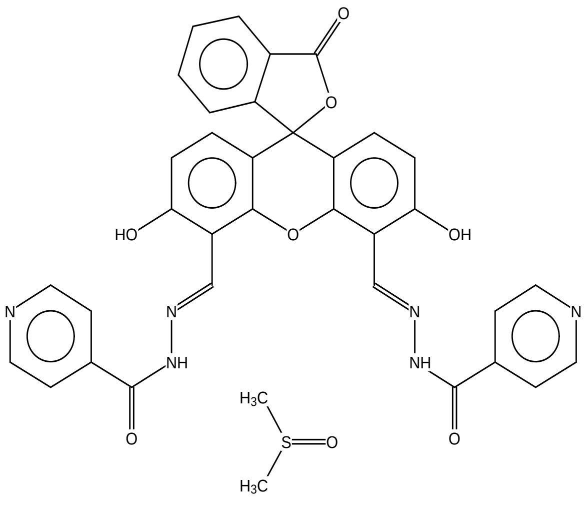

Search: search3 (Wed Apr 10 17:32:20 2019): Hits 21-23

WEVXOL

Reference: M.A.Peralta, M.N.V.de Souza, S.M.S.V.Wardell, J.L.Wardell, J.N.Low, C.Glidewell (2007) *Acta Crystallogr.,Sect.C:Cryst.Struct.Commun.* ,**63,**o68

Formula: $C_{15} H_{15} N_3 O_3, C_1 H_1 Cl_3$

Compound Name: 2,3-Dimethoxybenzaldehyde isonicotinoylhydrazone chloroform solvate

Space Group:	P21/n	***Cell:***	***a*** 12.740(0)	***b*** 10.860(0)	***c*** 13.919(0)		
Space Group No.:	14	***(Å,°)***	α 90.00	β 110.73(0)	γ 90.00		
R-Factor (%):	4.09	***Temperature(K):***	120	***Density(g/cm³):***	1.492		

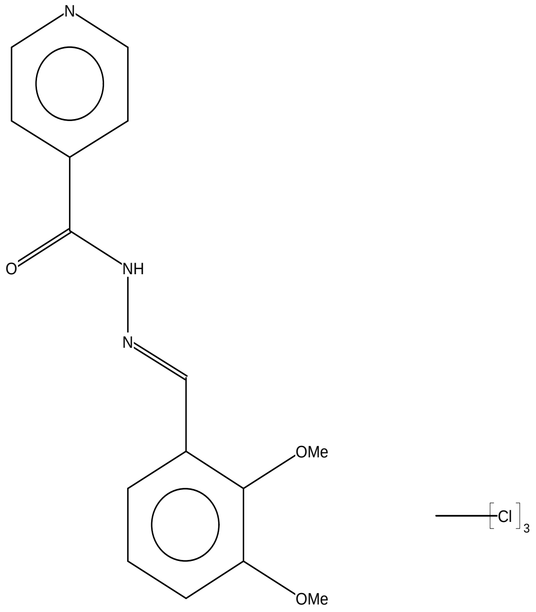

XIMGIK

Reference: Zhongwu Fu, Shaokang Gao, Shixiong Liu (2007) *Acta Crystallogr.,Sect.C:Cryst.Struct.Commun.* ,**63,**m459

Formula: $C_{28} H_{34} Cd_1 N_6 O_8{}^{2+}, 2(N_1 O_3{}^{1-})$

Compound Name: Tetra-aqua-bis(N-(2-methoxybenzylidene)pyridine-4-carbohydrazide)-cadmium(ii) dinitrate

Space Group:	P21/n	***Cell:***	***a*** 8.062(5)	***b*** 17.955(8)	***c*** 11.853(6)		
Space Group No.:	14	***(Å,°)***	α 90.00	β 98.26(2)	γ 90.00		
R-Factor (%):	2.78	***Temperature(K):***	293	***Density(g/cm³):***	1.602		

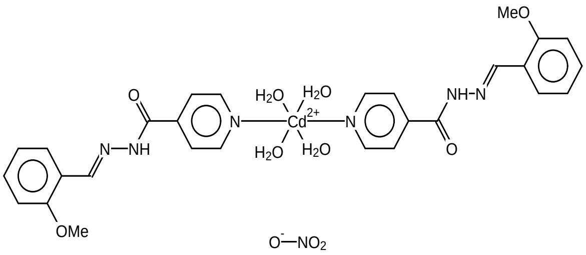

YIQFOU

Reference: S.M.S.V.Wardell, M.V.N.de Souza, J.L.Wardell, J.N.Low, C.Glidewell (2007) *Acta Crystallogr.,Sect.B:Struct.Sci.* ,**63,**879

Formula: $C_{15} H_{15} N_3 O_2$

Compound Name: N-isonicotinoyl 2-ethoxybenzaldehydehydrazone

Space Group:	P21/c	***Cell:***	***a*** 8.379(0)	***b*** 18.245(0)	***c*** 17.870(0)		
Space Group No.:	14	***(Å,°)***	α 90.00	β 90.98(0)	γ 90.00		
R-Factor (%):	5.59	***Temperature(K):***	120	***Density(g/cm³):***	1.310		

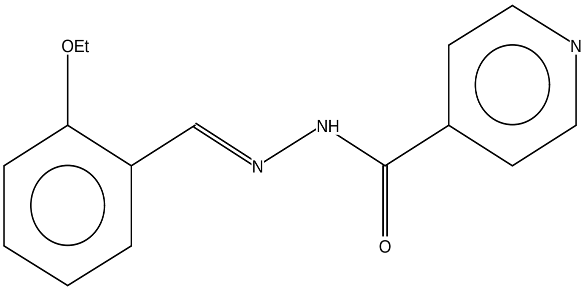

Supplement: Supplementary file 7 [file e-75-00655-sup7.pdf]
